# Stimuli-Responsive Langmuir Films Composed of Nanoparticles Decorated with Poly(*N*-isopropyl acrylamide) (PNIPAM) at the Air/Water Interface

**DOI:** 10.1021/acsomega.3c01862

**Published:** 2023-05-31

**Authors:** Rafał Zbonikowski, Michalina Iwan, Jan Paczesny

**Affiliations:** Institute of Physical Chemistry, Polish Academy of Sciences, Kasprzaka 44/52, 01-224 Warsaw, Poland

## Abstract

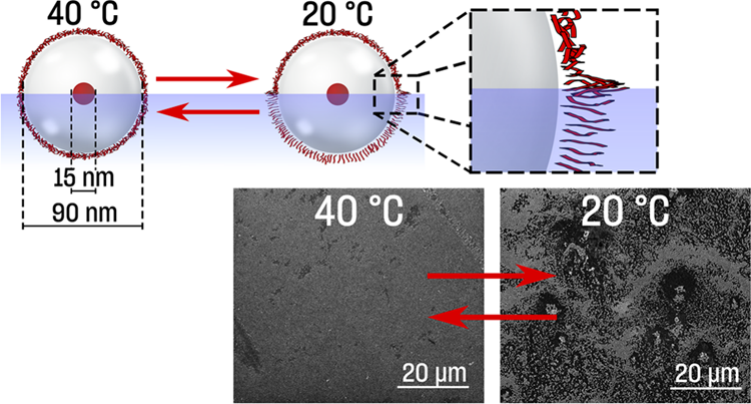

The nanotechnology shift from static toward stimuli-responsive systems is gaining momentum. We study adaptive and responsive Langmuir films at the air/water interface to facilitate the creation of two-dimensional (2D) complex systems. We verify the possibility of controlling the assembly of relatively large entities, *i.e.*, nanoparticles with diameter around 90 nm, by inducing conformational changes within an about 5 nm poly(*N*-isopropyl acrylamide) (PNIPAM) capping layer. The system performs reversible switching between uniform and nonuniform states. The densely packed and uniform state is observed at a higher temperature, *i.e.*, opposite to most phase transitions, where more ordered phases appear at lower temperatures. The induced nanoparticles’ conformational changes result in different properties of the interfacial monolayer, including various types of aggregation. The analysis of surface pressure at different temperatures and upon temperature changes, surface potential measurements, surface rheology experiments, Brewster angle microscopy (BAM), and scanning electron microscopy (SEM) observations are accompanied by calculations to discuss the principles of the nanoparticles’ self-assembly. Those findings provide guidelines for designing other adaptive 2D systems, such as programable membranes or optical interfacial devices.

## Introduction

Nature inspires scientists. Modern science goes further into the process than just adapting the solutions developed by nature. We study and utilize living systems and design artificial systems that are adaptive and stimuli-responsive. Dynamic self-assembly (DySA), which creates an out-of-equilibrium system requiring energy supply to persist, can be executed in a synthetic and beforehand planned manner.^1^ Although DySA wends toward its peak of interest,^2−9^ it is only another step in creating complex systems called chemical networks. Such networks are ubiquitous in living matter and can lead to a new chemistry age.^5^ This gives promise for nanotechnology to deliver on expectations, leading to nanomachinery’s next technological revolution.

Our recent review described a “stimuli-responsive material” as one that reacts to external stimuli, irreversibly transitioning from one local minimum to another or switching between different equilibria due to changes in parameters defining those equilibria. “Dynamic systems” require external stimuli, pushing them out of equilibrium. “Adaptive materials” present particular responses to given external stimuli and transition between equilibrium states, equilibrium and nonequilibrium states, or two or more nonequilibrium states.^10^ The review also gives a contemporary overview of adaptive two-dimensional (2D) and pseudo-2D systems.

The importance of colloidal assembly and the design of thin films was recently underlined by Vogel *et al*.^11^ The control over order/disorder was highlighted and presented as the fields to be developed. The authors stated that “Dynamic aspects of colloidal material will need to be increasingly considered, which carry tremendous potential for the development of advanced materials”.^11^

The number of reports describing interfacial adaptive and dynamic systems is slowly increasing.^12−15^ Martínez-Pedrero *et al*. reviewed and reported static and dynamic behavior of magnetic particles at fluid–fluid interfaces.^16−18^ Sashuk *et al*. showed the nanoscopic system, where amphiphilic nanoparticle aggregation was controlled by adding or removing the organic solvent to/from the interface.^12^ Klajn *et al*. presented light-induced aggregation of AuNPs decorated with azobenzene derivatives within the thin film of syndiotactic poly(methyl methacrylate).^14^ Such a system performed short-term, self-erasing information storage due to the relaxation and disaggregation. Similar properties of azobenzene building blocks were used by Vialetto *et al*.^15^ A quick-responsive 2D system of NPs crystallized under UV irradiation, presenting a highly organized structure.

Here, we present the stimuli-responsive 2D system of PSiFe nanoparticles, *i.e.*, Fe*_x_*O*_y_*@SiO_2_ nanoparticles with poly(*N*-isopropyl acrylamide) (PNIPAM) grafted on the silica surface. We investigated the dynamic and responsive properties of PSiFe within Langmuir and Langmuir–Blodgett films. We aimed to study the system controlled by several external stimuli, namely, magnetic field, mechanical stress, and temperature. In this article, we focused on the two latter factors governing the behavior of PSiFe films. Results showing the control of the particles in the magnetic field were inconsistent (poorly reproducible) and are described in the Supporting Information. Surprisingly, we found that a relatively thin polymer capping layer (around 5 nm) is controlled the behavior of rather large particles (around 90 nm in diameter). The thickness of the capping layer was around 20 times smaller compared to the diameter of the Fe*_x_*O*_y_*@SiO_2_ core. The importance of the properties and chemical composition of organic ligands capping nanoparticles was recently underlined.^19^

PNIPAM is known for its coil-to-globule (hydrated to dehydrated form) transition around 32 °C. The transition temperature is termed lower critical solution temperature (LCST). Below the transition temperature, the polymer creates hydrogen bonds with water molecules, while above the LCST, inter- or intramolecular hydrogen bonds prevail.^20^ The behavior of such polymers at fluid interfaces was recently described.^21,22^ PNIPAM-decorated magnetic particles have already been reported in different contexts, mainly in three-dimensional (3D) systems.^23−25^ These reports focus on aggregation properties or drug delivery potential, proving at the same time the temperature-dependent polymer transition on such nanoparticles. There is also a relatively large data set on PNIPAM-decorated graphene or graphene oxide and their applications.^26−28^ Recently, PNIPAM-capped SiO_2_ particles were shown as a promising adaptive material with tunable mechanical properties.^29^

Rezende *et al*.^30^ and Shan *et al*.^31^ studied 2D systems composed of PNIPAM-capped nanoparticles. Rezende *et al*. described the thermoresponsive monolayer properties of PNIPAM-decorated *ca*. 3.4 nm AuNPs,^30^ whereas Shan *et al*. studied a similar system with shorter and mixed polymer shell (PNIPAM and PS).^31^ Their research focused on the optical properties of such nanoparticles in relation to PNIPAM conformation. Stefaniu *et al*. presented a series of articles concerning 6.4 nm Fe_3_O_4_NPs capped with around 5 nm thermoresponsive polymers (random copolymer of 2-(2-methoxy ethoxy)ethyl methacrylate and oligo(ethylene glycol)methacrylate) or 2-(2-methoxy ethoxy)ethyl methacrylate) at the air/water interface.^32−36^ We analyzed their findings and discussed them with respect to our results.

We underline three main aspects of PSiFe thin films in this work. First is the responsive nature of the studied system, which might be considered a prototype of control switches for future chemical networks. Surprisingly, more uniform layers required heat influx to persist, unlike other self-assembly systems, where cooling down allows for ordering (*e.g.*, crystallization). Second, we present a strategy for controlling thin films’ morphology at the air/water interface and the solid substrates. Thin films are already widely used, and studying their principles should boost the development of areas such as photonics,^37^ spintronics,^37^ or sensors.^38^ Finally, we analyze the parameters governing the system. Interfacial phenomena,^39−42^ interparticulate interactions,^43^ and thermodynamics^44,45^ need to be precisely described for the rational design of dynamic and responsive systems. Understanding the nanoparticles’ immersion, conformation, aggregation processes, and interdigitation of the ligands allows for concluding the interactions between the nanoparticles. We believe the results presented here will help design future PNIPAM-based systems and nanoparticulate interface devices.

## Experimental Section

All chemicals were purchased from Sigma-Aldrich (Poznań, Poland) and used without further purification. Fe*_x_*O*_y_* seed particles were obtained from Siliquan (Warsaw, Poland). Organic solvents (chloroform, tetrahydrofuran (THF), hexene, ethanol) were at least of analytical grade. Ultrapure water (Milli-Q) was used.

### Design of PSiFe Nanoparticles

According to Tucker and Stevens,^46^ a single, isolated PNIPAM oligomer performs a different temperature transition depending on its length. They studied oligomers having 18 and 30 monomers (18 and 30 N). Short chains (shorter than or equal to 18 N) do not adapt a coiled form but remain extended at temperatures below the LCST. The temperature increase above the LCST results in partially folded conformation. Chains of at least 30 N could be coiled below the LCST. Above the LCST, the globule form is allowed. The tacticity of the polymers does not significantly influence the transition temperature of oligomers (as opposed to long PNIPAM chains).^46^

Shan *et al*.^47^ showed that the LCST changes upon oligomers’ attachment to the nanoparticle’s surface. Intermolecular (interligand) interactions occur at high concentrations, leading to a lower LCST than separated oligomers. For grafted chains shorter than 18 N, the LCST is much lower (it drops with the reducing molecular weight) than for longer oligomers and polymers, both free or attached to a surface (LCST is close to 30–32 °C). On the contrary, the LCST of free NIPAM oligomers rises with reducing the molecular weight even above 60 °C.

Based on these reports, we judiciously designed the studied PSiFe particles. First, we wanted to observe the coiled-to-collapsed transition of PNIPAM upon the temperature change. This required that chains should not be too short. Therefore, the oligomers should have more than 18 N, as such chains could only bend above the LCST. However, the chains should not be too long, as we aimed at extended conformation below the LCST. We hypothesized that this would facilitate the nanoparticles’ interactions below the LCST. Therefore, chains shorter than 30 N were required (longer chains tend to coil). Thus, we used PNIPAM of the average mass of 2750 g·mol^–1^, and the polydispersity index (*M*_w_/*M*_n_) was below 1.8. Based on this, the number of monomers in the ligand could vary from around 20 to 29 N. The maximum of the distribution was around 24 N. Such a choice also justifies the two experimental temperature regimes, *i.e*., 20 and 40 °C, leveling the polymer’s LCST in the middle of the experimental range with reasonable offsets (around 10 °C).

A similar length of PNIPAM grafted on the gold nanoparticles was already tested, but the cores’ diameter was much smaller (in the range of a few nanometers).^31,47^ It was shown that the PNIPAM layers could not overlap when attached to small gold cores.^31^ There were two main differences between previous reports and the studied system. First, in our work, ligands were well-anchored to the oxide layer (typical energy of the Si–O bond is around 100 kcal·mol^−1^) and each PNIPAM ligand might form up to three such bonds with the surface^48^ in contrast to the Au–S bond (commonly reported values are between 20 and 60 kcal·mol^−1^)^49^ character, which allows for ligand rearrangements at the surface of the nanoparticles.^50^ Second, we attempted to show the possibility of controlling the self-assembly of relatively large objects by relatively small ligands.

### Synthesis of Fe*_x_*O*_y_*@SiO_2_ Nanoparticles

99.8% ethanol (30 mL, 23.348 g), distilled water (3.0 mL, 2.999 g), and 23% aqueous solution of ammonia (1.5 mL, 1.487 g) were added to a plastic vial. Then, 1 mL of Fe*_x_*O*_y_* Siliquan (Warsaw, Poland) nanoparticles with a 15 nm diameter was injected into the mixture as it was stirred at room temperature. After 15 min, tetraethyl orthosilicate (TEOS, 150 μL) was added, and the sample was sealed. The mixture was stirred at room temperature for 24 h. The concentration of the obtained nanoparticles was 1.89 mg·mL^–1^.

### Functionalization of Fe*_x_*O*_y_*@SiO_2_ Nanoparticles

The PNIPAM polymer was purchased in the form of a triethoxysilane derivative. *M*_n_ was 2750 g·mol^–1^, and the polydispersity index (*M*_w_/*M*_n_) was below 1.8.

8.40 mg of PNIPAM derivative was dissolved in 99.8% ethanol (3 mL). The polymer solution was added to a glass vial containing 30 mL of the obtained Fe*_x_*O*_y_*@SiO_2_ nanoparticles at 28 °C. The reaction mixture was stirred at 28 °C for 72 h.

### Purification of PSiFe

To the Fe*_x_*O*_y_*@SiO_2_@PNIPAM mixture, hexane (30 mL) and 99.8% ethanol (18 mL) were added, giving a homogeneous solution. The nanoparticles were centrifuged at 9000 rpm for 45 min. The supernatant was discarded, and ethanol (10 mL) was added to the remaining lightly orange precipitate. The solution was sonicated for 5 min, hexane (20 mL) was added, and samples were centrifuged at 9000 rpm for 30 min. The purification procedure was repeated four times, discarding the supernatant after each centrifugation. After the last centrifugation, distilled water (9.9 mL) was added to the precipitate, allotting a concentration of 5 mg·mL^–1^ of nanoparticles. The solution was sonicated for another 30 min. Subsequently, it was left in an open vial at room temperature for 30 min to allow any hexane residues to evaporate before sealing the vial.

The characterization of the obtained PSiFe particles is given in the Supporting Information (Figures S1–S3, Table S1).

### Sample Preparation

The purified suspension of PSiFe nanoparticles in water (5 mg·mL^–1^) was stored in a fridge. It was diluted with THF in a volumetric flask to get the final concentration of 1 mg·mL^–1^. The suspension was mixed, sonicated for a few minutes, and stored at room temperature. Before the experiment, the sample was sonicated for at least 2 min prior to deposition onto the air/water interface.

### Trough Cleaning and Preparation

Before every experiment, Langmuir/Langmuir–Blodgett troughs were cleaned with chloroform, ethanol, and ultrapure water. Then, they were left to dry. The interface was cleaned using a Teflon sucking nozzle. The cleaning procedure was repeated until the surface pressure increase upon reducing surface area from maximal to minimal was not higher than 0.1 mN·m^–1^. A NESLAB RTE 7 thermostat was used to keep the system at the desired temperature.

### Deposition of PSiFe at the Air/Water Interface

The subphase with the prepared interface was set to 40 °C (±2 °C) with the barriers in the fully open position. For the deposition of PSiFe onto the interface, we used the method described before.^51,52^ In short, the clean glass rod was immersed in the central part of the trough. The desired amount of the sonicated sample was deposited dropwise using a Hamilton syringe. The droplets of PSiFe suspension were placed at the unimmersed part of the glass rod, just above the water surface. After the deposition, the glass rod was gently removed. The trough was left for 20 min to let the solvent evaporate. Next, the temperature of the subphase was decreased if needed.

### Langmuir Systems

Two different experimental setups were used for different types of experiments. Both were placed in Plexiglas boxes and on the passive antivibration tables. The compression experiments were performed on both Langmuir troughs to verify the reproducibility of the results. We provide information on which the set was used to obtain the results presented in the publication (NIMA *versus* KSV NIMA).

NIMA: 375 cm^2^ trough, Teflon barriers, paper Wilhelmy plate surface pressure sensor, KEITHLEY 196 system DMM with a Pt100 temperature sensor, optionally MiniBAM, Kelvin electrode, dipper.

KSV NIMA: 835 cm^2^ trough, Teflon barriers, paper Wilhelmy plate surface pressure sensor, default temperature sensor, optionally Kelvin electrode, dipper, KSV NIMA MicroBAM.

In the case of prolonged experiments in which the evaporation of water from the subphase was an issue, we controlled the relative position of the interface with respect to the Wilhelmy sensor. First, all experiments were done in closed boxes, which protected the films from contaminants and the environment (*e.g*., air currents) and allowed us to control the humidity. We introduced saturated vapors when necessary or performed a control experiment without nanoparticles and subtracted the evaporation background from the isotherms. We replenished the water manually when the same film was compressed many times but with long intervals before the experiments.

### Stability Experiment, Isotherms of Compression, and Surface Potential Measurements

The experiments were performed using the NIMA set with a Kelvin electrode. 500 μL of the sample was deposited. After about 20 min, one compression and decompression cycle was performed with a barrier speed of 10 mm·min^–1^. Then, consecutive portions (250 μL) were added, and the procedure was repeated until getting the final isotherm for 1000 μL of the sample. The measurements were performed in an analogical way for 40 and 20 °C. Surface potential was measured simultaneously with surface pressure.

### Stimuli-Responsive Langmuir Isotherms—Experiments

The experiment was conducted using the NIMA set and confirmed with the KSV NIMA set. 500 μL of the sample was deposited on the interface at 40 °C and left for 20 min to equilibrate. Compression and decompression cycles were carried out at the rate of 10 mm·min^–1^. Between the first and the second run, the time gap lasted around 2 h, the second and the third—18 h, the third and the fourth—2 h. The water level was checked before each compression. MiniBAM was used and aimed onto the most central position allowed.

### Elasticity and Viscosity Measurements

Surface compressional modulus was calculated based on the compression isotherms. We used the oscillatory barrier method to find interfacial dilatational elastic modulus and interfacial dilatational viscous modulus. The NIMA set was used. PSiFe nanoparticles (500 μL of the sample) were compressed (10 mm·min^–1^) to the surface pressure ∼6.5 mN·m^–1^. The system was equilibrated for 60 min, and the barrier oscillations started with a specific frequency ranging from 0.015 to 0.209 Hz. Ten cycles of the area amplitude of 2.5 cm^2^ were measured. This corresponded to up to around 5 to 10% of the surface area of the film. This was allowed because of the unusual geometry (*i.e.*, length-to-width ratio) of the used NIMA set.^53^

### Relaxation Experiments

The KSV NIMA system was used for the relaxation experiment. 1500 μL of nanoparticles was deposited at 40 °C and then left for approximately 20 min to let the solvent evaporate. Then, the system was equilibrated at 40 °C (around 20 min) or 20 °C (around 2 h, as it required cooling down after PSiFe deposition). Nanoparticles were compressed (10 mm·min^–1^) to the surface pressure of around 11 mN·m^–1^. The barriers were stopped immediately after reaching this value, and the surface pressure was measured for at least 2 h.

### Transfer of the Monolayer onto a Solid Substrate

The KSV NIMA system with a dipper was prepared as described before. The silicon wafers were used as a solid substrate. Wafers were cleaned with 30% nitric acid, distilled water, ethanol, and acetone. The solid substrate was immersed vertically at the center of the trough before the compression. The nanoparticle film was compressed (10 mm·min^–1^) to the target pressure and left to reach the equilibrium for 25 min. Then, the substrate was pulled out at the rate of 13 mm·min^–1^.

### Other Instrumentation

A Nova NanoSEM 450 was used for the scanning electron microscopy analysis.

Dynamic light scattering (DLS) and ζ-potential measurements were carried out using a Zetasizer Nano ZS apparatus (Malvern Instruments Ltd., Malvern, U.K.) equipped with a DLS module (He–Ne laser 633 nm, max 4 mW, allowing for measuring backscattered (175°) and forward-scattered (12.8°) light). Quasi-backscattered light was used for the measurements.

## Results and Discussion

In experimental data and discussion, HT (high/higher temperature) refers to the temperature 40–42 °C, while LT (low/lower temperature) means 18–20 °C. Those regimes correspond to above or below the LCST, *i.e.*, “closed” and “open” PNIPAM capping ligands, respectively.

### Langmuir PSiFe Films

There was no apparent asymmetry in the structure of the particles to provide amphiphilic character, usually required in Langmuir–Blodgett studies. Our previous work showed that the necessary balance might be assured by differences in interactions between ligands and cores with the water molecules.^54^ Also, Guzmán *et al*. described the particles laden at the interfaces.^55^ The case of PSiFe was challenging as particles formed suspension in water. For the particles to remain at the interface, we established the procedure of the material deposition. This required proper cosolvents and gentle application of the PSiFe suspension (*cf*. Experimental Section). Next, we evaluated whether PSiFe forms stable Langmuir films. First, the isotherms showed hysteresis while performing the compression–decompression cycle, *i.e.*, they were poorly reversible (Figure S4). At HT, consecutive compression isotherms were reproducible, with only minor shifts of consecutive compression isotherms. The significant shifts of isotherms toward smaller values of surface area were observed at LT (Figure S4). This suggested irreversible aggregation upon compression at LT.

**Figure 1:**
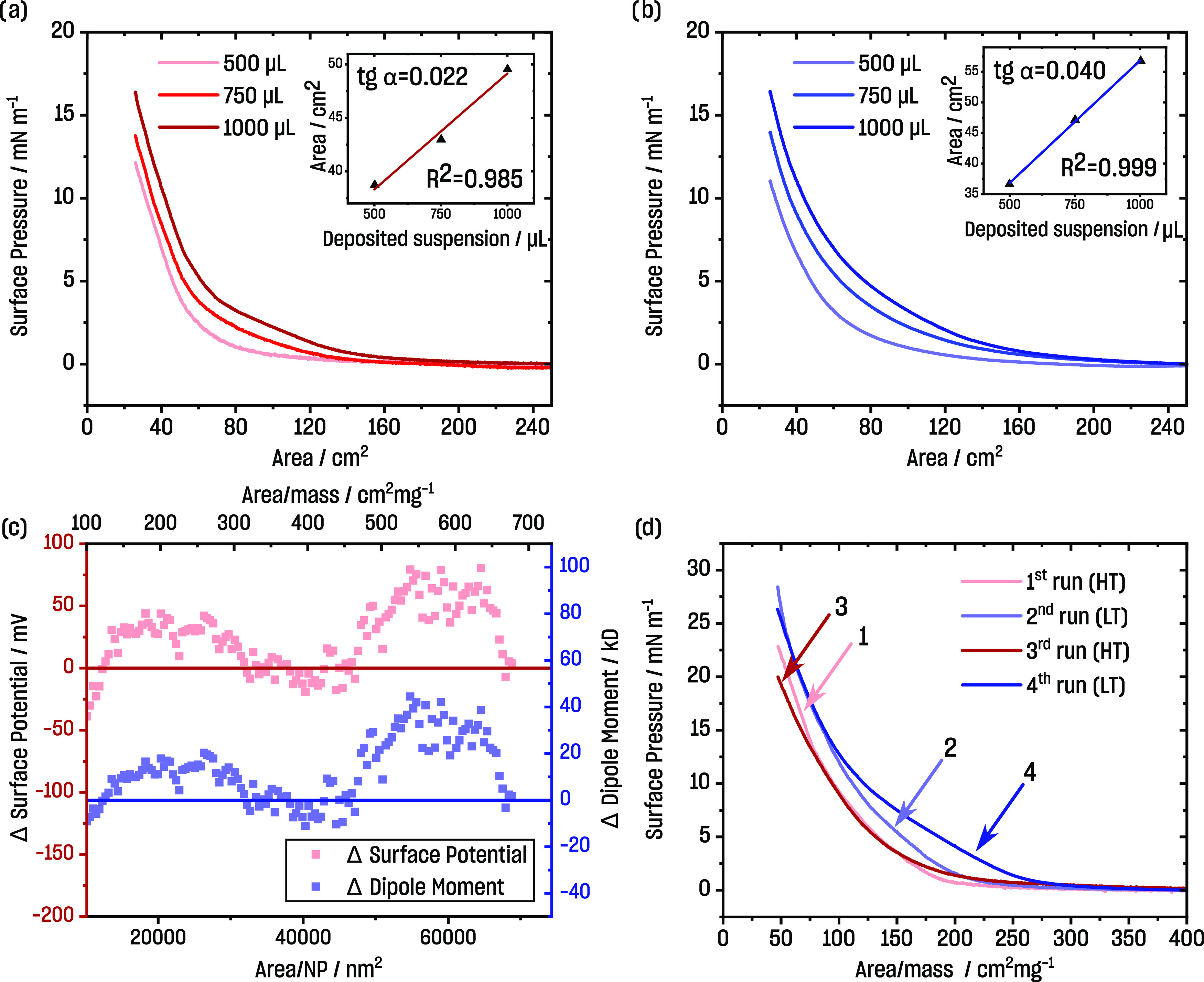
Consecutive compression isotherms upon the addition of aliquots of PSiFe nanoparticles onto the air/water interface at (a) HT (40–42 °C) and (b) LT (18–20 °C). The insets show linear dependence between surface areas at π = 7.5 mN·m^–1^ and the deposited volume of PSiFe. (c) The differences (LT minus HT) in the surface potential and dipole moment were calculated according to the Helmholtz equation (eq 1). (d) Isotherms of compression showing the switchability of the PSiFe nanoparticle monolayer upon consecutive temperature changes between HT and LT. The isotherms of decompression were ignored in graphs (a), (b), and (d) to keep the clarity of the figures. Hysteresis within compression–decompression cycles can be found in the Supporting Information (Figures S4 and S6).

Next, we deposited subsequent portions of nanoparticles onto the interface in between compression cycles. The addition of the solvent was aimed to facilitate the spreading of any preformed aggregates. The gradual, almost linear change of the area at constant surface pressure (7.5 mN·m^–1^) occupied by the increased amount of particles was observed (see insets in Figure 1a,b). Only slight deviations were noticed when expressing the isotherms in cm^2^·mg^–1^ (*e.g.*, normalized concerning the amount applied at the interface; Figure S5a,b). These small shifts toward smaller surface areas originated in the nonperfect spreading of particles upon the consecutive additions of PSiFe, as shown by BAM observations (*cf*. Figure 3). The linear dependence between the surface area and amount of deposited particles showed that the films had 2D character. We expected a proportional increase of surface area with applied volume, but some fraction of particles were lost (either drowned or aggregated) during the experiment. This was in line with a small shift in the isotherms’ positions. Nonetheless, the developed protocol was adequate for studying PSiFe at the air/water interface using the Langmuir–Blodgett technique when controlling for the application procedure, the amount of applied PSiFe, and the concentration of PSiFe stock.

Compression of PSiFe particles resulted in isotherms showing a monotonic surface pressure increase. We were not able to reach the apparent collapse even when we applied 1.5 mL of 1 mg·mL^–1^ PSiFe suspension. For very high volumes of PSiFe applied and high compression, we observed the formation of multilayered films but without the kink at the isotherms (*cf*. SEM picture analysis section in the Supporting Information). We restricted our study to low values of surface pressure as the system was designed to work and adapt to stimuli within a monolayer. We aimed to avoid uncontrolled phenomena occurring upon collapse.

More details justifying the used procedure can be found in the Supporting Information (*i.e*., trapping energy,^39,55−60^ temperature gradient analysis^61^).

When comparing identical conditions with exemption to temperature, the shift toward a higher surface area per nanoparticle was visible for LT in comparison to HT. In the case of non-thermo-responsive particles or molecules, the effect is opposite due to thermal expansion, *i.e.*, the same film compressed to the same surface pressure but at a higher temperature occupies a larger surface area, and the isotherm is shifted to the right^62^ (*cf*. Isotherms of non-thermo-responsive materials section in the Supporting Information(63)).

In addition to surface pressure, the surface potential was simultaneously measured (Figure S5). The surface potential, Δ*V*, is related to the vertical component of the molecular dipole moment by the Helmholtz equation^64^
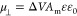
1where μ_⊥_ is the average component of the molecular dipole moment normal to the plane of the monolayer, *A*_m_ is the area per particle, and ε and ε_0_ are dielectric permittivity constants of the monolayer and vacuum, respectively.

The surface potential isotherms are shown in the Surface potential and dipole moment section in the Supporting Information (Figure S6). Below the LCST (LT), the growth of the surface potential occurred at a higher area/mass ratio than HT. For instance, at 150 cm^2^·mg^–1^, the surface potential at LT was around 230 mV, whereas at HT, only around 160 mV. The same relation was observed when the same surface pressure was considered.

The difference between the surface potential and dipole moment at LT and HT is shown in Figure 1c. The biggest differences between LT and HT were observed at the surface area much larger than the onset of the surface pressure isotherm (above 450 cm^2^·mg^–1^), *i.e.*, at low surface concentrations of PSiFe. At around 350 to 400 cm^2^·mg^–1^, the difference was close to zero. Further compression (below 300 cm^2^·mg^–1^) resulted again in higher values of the surface potential at LT compared to HT.

Not many reports focus on the temperature’s influence on the surface potential. In one example, Nakahara *et al*.^62^ studied a mixture of dipalmitoylphosphatidylcholine (DPPC)/egg-phosphatidylglycerol (PG)/palmitic acid (PA) and amphiphilic R-helical peptide (Hel 13–5) or commercially available surfactant (Surfactant TA). The surface potential values decreased, and the onset shifted toward values of the smaller surface area with the increase in temperature. Such a decrease was likely related to a more disordered arrangement of molecules within films at the higher temperature. We previously showed such “loosening” of the structure in the case of hyperbranched polymers.^65^

### Stimuli-Responsive Langmuir Isotherms

**Figure 2:**
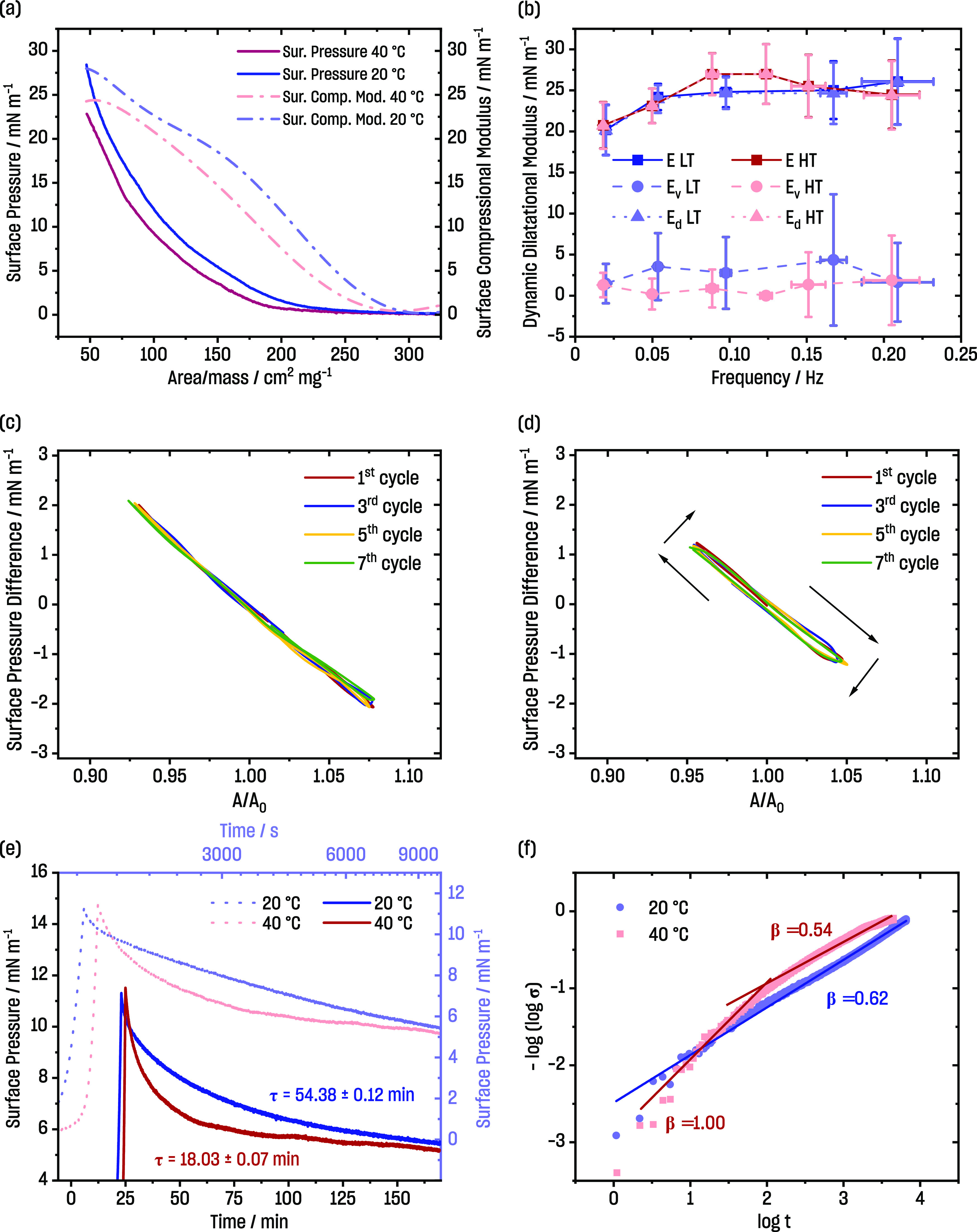
(a) Surface pressure isotherms at different temperature regimes (HT—40 °C and LT—20 °C) with corresponding surface compressional modulus; (b) results of surface dilatational elasticity modulus (*E*—complex surface elasticity, *E*_*v*_—dilatational viscosity, *E*_*d*_—surface dilatational elasticity); (c) Lissajous diagram for the PSiFe film at HT (0.10 Hz); (d) Lissajous diagram for the PSiFe film at LT (0.10 Hz); (e) relaxation of the PSiFe monolayer after the compression to 11 mN·m^–1^ with time expressed in linear (vivid colors) and logarithmic scales (pale colors), and (f) values of β parameters, which were calculated from relaxation curves, were related to interactions within the film.

The possibility of reversible and controlled changes in nanosystems and processes is crucial for creating adaptive systems and chemical networks.^5^ The studied system performed reversible switching between two states. First, the compression isotherms were measured upon temperature changes from 40 to 20 °C and back. For the lower temperature (≈20 °C), the curves were shifted toward higher surface areas compared to high temperature (≈40 °C) (Figure 1d). This was in line with compression isotherms obtained without the temperature changes during the experiment (Figures 1a,b and 2b) and opposite to non-thermo-responsive films (*cf*. Isotherms of non-thermo-responsive materials section in the Supporting Information). The isotherms were reproducible at HT. Some irreproducibility was observed at LT. The second heating cycle (third isotherm, HT) resulted in the better spreading of PSiFe at the interface, causing the difference between the second and fourth isotherms at LT. The deviations between isotherms at LT were observed only at low surface pressure values, most likely originating in the kinetic effect as described by Kwok *et al*.^66^ The isotherms at LT overlapped at low surface areas (high surface pressure), which differentiated this experiment from consecutive compressions at LT, without intermediate heating to HT (*cf*. Supporting Information, Figure S4).

### Rheological Measurements: Surface Compressional Modulus

The surface compressional modulus was calculated according to the equation
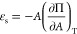
2where ε_s_ is the surface compressional modulus, *A* is the area per nanoparticle, and Π is the surface pressure. The surface compressional modulus represents the change of surface pressure concerning the relative change of the surface area. ε_s_ is often used to characterize the monolayer properties. Its values correspond to the film’s state, being greater for more condensed films.^67^

For the whole compression experiment, the values of surface compressional modulus curves recorded at LT were higher those at HT (Figure 2a). The maximal observed values of surface compressional moduli were around 20 to 30 mN·m^–1^. Such values are usually considered relatively low, and for amphiphiles, they correspond to the liquid expanded phase.^67^

There was no apparent maximum in the surface compressional modulus curves upon compression. The maximum in surface compressional modulus curves usually appears before the collapse. Therefore, not reaching the maximum of the compressional modulus meant that we did not reach the apparent collapse of the films either. This was in line with our observations that, for a high amount of PSiFe deposited onto the interface and high values of surface pressure, the film became multilayered continuously, without sudden disruption of the 2D structure (*cf*. SEM picture analysis section in the Supporting Information). This again suggested that the switching operation of our system should be studied at relatively low surface pressures.

### Rheological Measurements: Surface Dilatational Elasticity Modulus

Usually, the ideal compression–decompression cycle of the insoluble Langmuir monolayer should be conducted for |*v*| → 0 (*v*—barrier velocity), *i.e*., conditions as close to equilibrium as possible. However, dynamic analysis can be utilized to study the properties of thin films. Therefore, viscoelastic characteristics were measured using the oscillating barrier method. PSiFe thin films underwent small compression/decompression cycles (changes in the surface area were up to around 5 to 10%) with a specific frequency.^68,69^ We showed that the experiments were done in the linear regime (*cf*. Supporting Information, Figure S7). The Supporting Information contains further analysis related to total harmonic distortion.^70,71^

The oscillating barrier method allowed for verifying how films respond to stress.^72^ The surface dilatational elasticity modulus is a complex quantity described by the equation
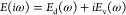
3*E*_d_ is the interfacial dilatational elasticity moduli, and *E*_v_ is the surface dilatational viscosity moduli, , where *f* is the frequency. *E* can also be written as .^69,73,74^ From this part, according to the trigonometrical properties, *E*_d_ = |*E*| cos θ and *E*_v_ = |*E*| sin θ,^69,74^ θ is the phase angle, *i.e.*, the delay between the imposed area deformation and the response in the surface pressure. The elastic properties were dominant in both temperature regimes and at all tested frequencies (*E*_d_ ≫ *E*_v_) (Figure 2b). Similar to the analysis of the surface compressional modulus, the surface dilatational elasticity modulus of PSiFe films showed a very similar behavior at both temperatures.

We prepared the Lissajous diagrams in accordance with the report by Morioka and Kawaguchi.^75^ The positive hysteresis (Figure 2c,d) observed for PSiFe at LT indicated that there was no piling up of the particles during experiments. The hysteresis was even more profound for 0.05 Hz (*cf*. Supporting Information, Figure S9). Increasing the temperature and causing PNIPAM change to the “close” structure resulted in the Lissajous diagrams without any hysteresis. The negative hysteresis was not observed.

It must be underlined that, despite 60 min of equilibration, there was a small drift in the surface pressure readout during the measurements (*cf*. Rheological measurements section in the Supporting Information). Without correcting it, the consecutive Lissajous diagrams were shifted. This had no effect on the “direction” of hysteresis in the Lissajous diagrams (marked with arrows). We also recalculated the surface dilatational elasticity modulus, and there were no significant differences between data corrected for the drift and without correction.

### Rheological Measurements: Relaxation Experiments

Rheological properties might also be elucidated from the relaxation experiments. First, the films were compressed to the target pressure. The target pressure was reached much faster at LT than at HT (Figure 2e). The experiments were performed at a constant speed of the barriers, which was the same at both temperatures, and thus, this corresponded to the reciprocal of the surface area. Therefore, the shift was due to the PNIPAM ligands being in the “open” conformation at LT, causing individual PSiFe particles to occupy a larger area than the “closed” conformation at HT. This was in line with the compression experiments described in the previous sections.

After reaching the target pressure, the films were allowed to relax. The relaxation runs gradually in both temperature regimes. As opposed to the surface compressional modulus and the surface dilatational elasticity modulus, relaxation experiments showed apparent differences in the properties of PSiFe films at LT and HT. The decrease of the surface pressure for the higher temperature in the first minutes was more significant.

We analyzed the data according to the method described by Hilles *et al*.^76^ First, we established that the relaxation time (function  was fitted) was much longer for LT *versus* HT (around 54 *versus* 18 min, respectively). The character of surface pressure curves as a function of the natural logarithm of time was different at LT and HT (upper *X*-axis in Figure 2e). It was shown before that a more linear decrease was due to stronger interactions between molecules at the interface (here LT), resulting in a more condensed phase when compared to a logarithmic decrease, often associated with liquid-like phases (here, HT).^65^ These observations supported that PNIPAM was in the “open” conformation at LT, allowing for more interactions between PSiFe particles compared to the “closed’ ligands at HT.

Next, we used the Kohlrausch–Williams–Watts function^76^
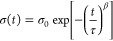
4where σ_0_is the amplitude of relaxation, τ is the time needed to reach the equilibrium, *t* is the relaxation time, and β is the parameter related to the interactions. The additional relations are σ_0_ = π_*t*0_ – π_∞_ and σ = σ_0_ – π_∞_, where π_*t*0_ is the surface pressure at the beginning of the relaxation and π_∞_ is the surface pressure at the equilibrium, which brings us to
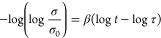
5We plot  as a function of log *t* to extract β. The analysis of the β parameter was inspired by Hilles *et al*.^76^ They said that, for β = 1, there was no coupling in the system, whereas β = 0.5 corresponded to “*high cooperative motions*”. The results for PSiFe are presented in Figure 2f. The value of β (0.62) at 20 °C suggested strong cooperativity.^76^ The relaxation was exponential. This was not true at 40 °C. At the beginning of the relaxation, the fitted β value was around 1—the boundary condition of the model (β = 1 means no coupling). The β parameter decreased but only at times longer than relaxation time (*i.e*., around 18 min). It needs to be noted that the theory used here was derived for polymers. We still argue that it gave an insight into the behavior of PSiFe films, as their behavior was governed by the interactions of polymeric, PNIPAM ligands.

### Brewster Angle Microscopy Observations

**Figure 3:**
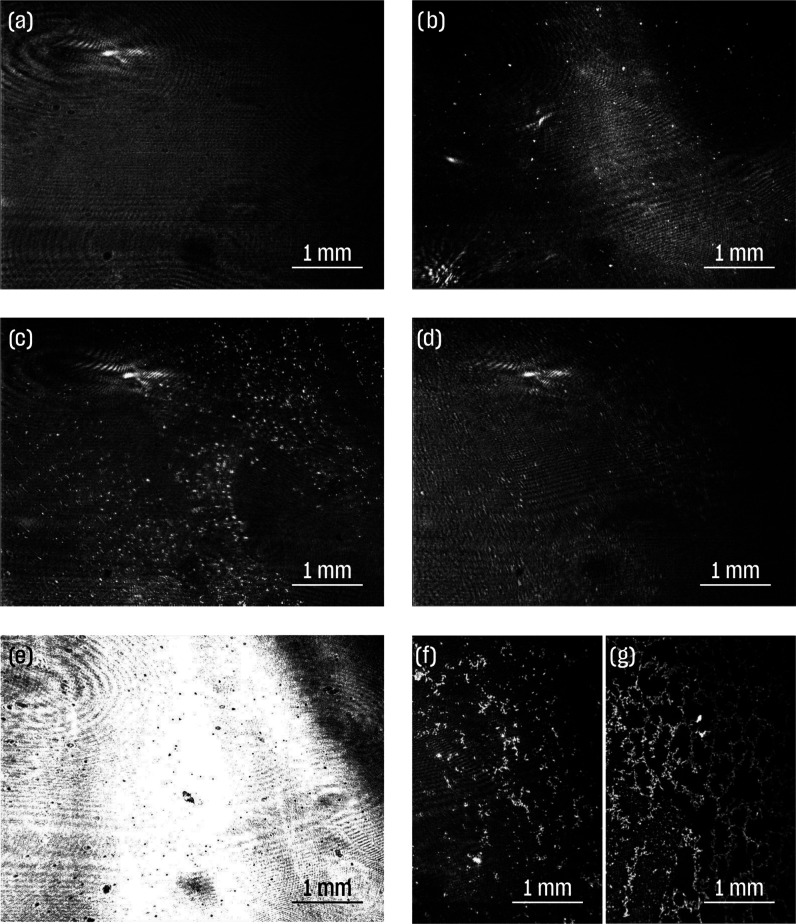
BAM pictures of PSiFe Langmuir films: (a) after deposition and relaxation at HT, (b) after deposition at HT and cooling down to LT, (c) upon compression at HT (122 cm^2^, 0.3 mN·m^–1^), (d) consecutive decompression at HT (126 cm^2^, 0.2 mN·m^–1^), (e) upon compression at LT (132 cm^2^, 1.9 mN·m^–1^; the picture was taken with the same brightness/contrast parameters, and its high brightness corresponds to the dense layer of the film), and (f) upon decompression at LT (142 cm^2^, 0.2 mN·m^–1^), with (g) a visible dendrimer-like network (LT).

Brewster angle microscopy allowed the direct observation of PSiFe films at the air/water interface during the compression and decompression. The observed features of films differed depending on the temperature. Above the LCST (HT), no distinctive features were visible upon the deposition of PSiFe at the interface (Figure 3a). The film was barely distinguishable even upon full compression, with only small bright aggregates (Figure 3c). These aggregates became less visible upon decompression (Figure 3d) and finally disappeared completely. Far below the LCST (LT), the film was visible before compression, *i.e.*, just after the deposition of the materials (Figure 3b). Upon compression, BAM pictures were very bright (Figure 3e). Decompression revealed the formation of characteristic fractal, dendrimer-like aggregates (Figure 3g) or a gradually disaggregating network (Figure 3f).

### Langmuir–Blodgett Films of PSiFe

**Figure 4:**
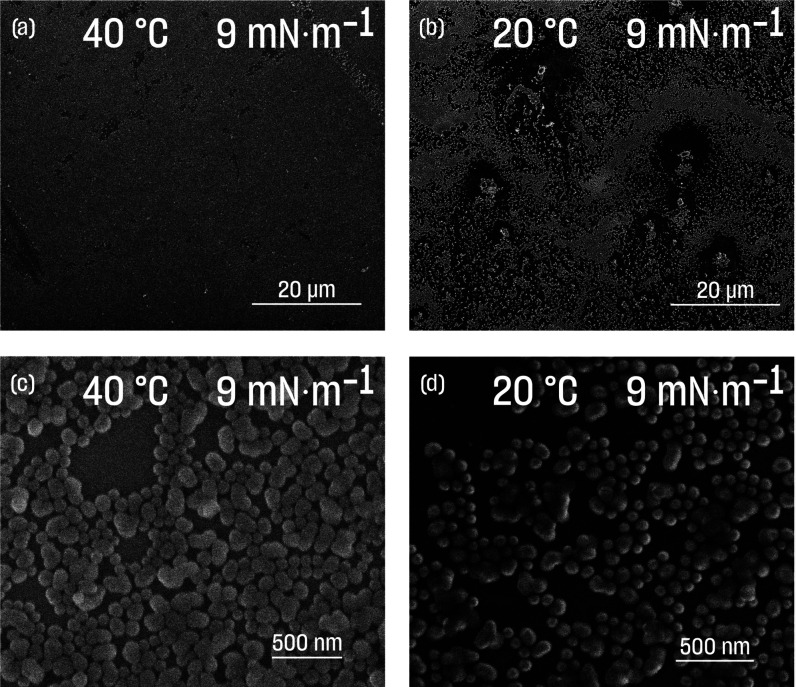
SEM pictures of PSiFe films transferred at the same surface pressure (9 mN·m^–1^) but at different temperatures, *i.e.*, at 40 °C (a, c) and 20 °C (b, d).

We transferred films onto the solid substrate at low and high temperatures but at the same surface pressure of 9 mN·m^–1^. Scanning electron microscopy (SEM) observation showed that films prepared at HT were densely packed and uniform (Figure 4a). Individual PSiFe particles were in proximity, often being in contact with neighboring particles (Figure 4c). On the contrary, films transferred at LT were not uniform but somewhat distorted, with evident variations in the surface density of PSiFe particles (Figure 4b). The most densely packed areas resembled those observed in high temperatures. Individual PSiFe particles were separated by the free spaces (Figure 4d). Additional analysis of SEM pictures clearly showed that the particles at LT were further apart (around 10 nm on average) compared to HT (below 5 nm on average) (Figure S11).

The decrease in temperature usually results in ordering (*e.g*., crystallization). Here, energy flux was needed to allow for the order within the films. The increased temperature was necessary to induce the conformational change of ligands, which governed the self-assembly process. This appeared possible, despite significant disproportions between the diameter of PSiFe cores and the thickness of PNIPAM capping layers.

We also performed SEM observations for samples deposited on the solid substrate at high surface pressure (25 mN·m^–1^). This showed the formation of multilayered films at high compression without the apparent collapse visible in the surface pressure curves. The pictures are provided in the SEM picture analysis section in the Supporting Information.

In the number of attempts, we were able to form anisotropic structures within PSiFe films using a magnetic field. However, these experiments were inconsistent and poorly reproducible, probably due to experimental limitations. The results are shown in the Magnetic properties section in the Supporting Information.

### Discussion

We rationalize the observed behavior of PSiFe films at LT and HT based on the differences in PNIPAM conformation. This is an example of relatively small capping ligands (below 5 nm) controlling large objects (particles around 90 nm in diameter).

We use the following terms: (1) extended: below the LCST (lower critical solution temperature), hydrated short chains (too short to coil); (2) coiled: below the LCST, long chains; (3) folded: above the LCST, short chains (too short to adapt the globule form), also interacting with each other at the NP surface; and (4) globule: above the LCST, long chains (typical for a suspension of long PNIPAM chains above the LCST). In general, the conformations below the LCST (*i.e*., extended and coiled) are termed “open”, and above the LCST (*i.e*., folded and globule) “closed”.

### PSiFe Films at the Air/Water Interface

We aimed to understand the behavior of PSiFe films upon compression and temperature changes. Shan *et al*.^31^ described three regions visible in the isotherms upon compression of 4.2 nm AuNPs stabilized with PNIPAM mixed with polystyrene (PS) (around 28 and 24 N, respectively). These regions corresponded to the conformational changes of PNIPAM upon compression and temperature change. However, the authors provided surface plasmon resonance (SPR) measurements only at temperatures 30 °C or lower. Therefore, the results might not be sufficient to observe fully closed nanoparticles above the LCST.

We observed a similar shape of the PSiFe compression isotherms as Shan *et al*.^31^ Still, our data suggested that the origin of the three regions might not directly come from the conformation changes of PNIPAM. It must be underlined that our nanoparticles were much larger than those studied by Shan *et al*.^31^ We observed similar shapes of the isotherms at both low and high temperatures. At HT (*cf*. Figure 1a), PNIPAM was above the LCST (“closed” conformation), and no conformational changes within the capping layer could occur upon compression. From this, we concluded that the shape of the isotherms reflected the behavior of the whole nanoparticles and was not directly related to PNIPAM ligands. Below the LCST (LT), PNIPAM conformational changes added complexity to the system but did not determine the isotherms’ appearance.

### Conformation and Immersion of PSiFe

**Figure 5:**
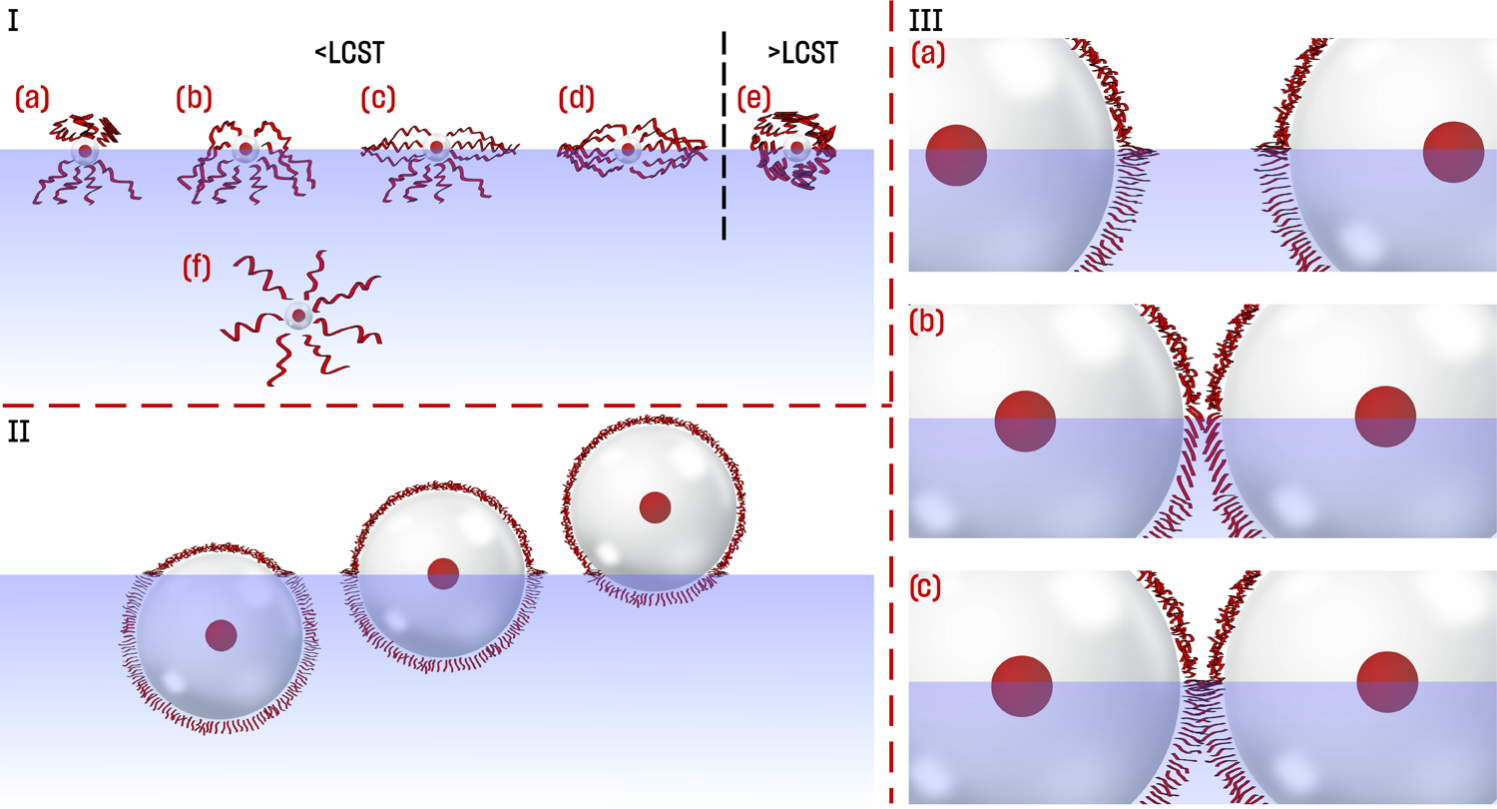
(I) Conformations of PNIPAM-capped nanoparticles proposed in the literature. These conformations were described for relatively small nanoparticles with relatively long PNIPAM chains attached. (a) “Janus” conformation, (b) “brush” conformation, (c) “jellyfish” conformation, (d) “pancake” conformation, (e) closed conformation, and (f) fully open, anisotropic conformation. Structures (a–c) are possible above and (d) below threshold surface pressure and only below the LCST. Conformation (e) exists above the LCST. (II) Scheme showing possible immersion of PSiFe nanoparticles’ below the LCST. (III) Scheme showing possible conformations of PSiFe nanoparticles at the air/water interface: (a) at low surface pressure, (b) at high surface pressure with the deflection of the polymer chains, (c) at high surface pressure with the interdigitation of the polymer chains. The capillary effect (if it exists in the system) was neglected in the figure.

The summary of possible conformations of PNIPAM at the surface of nanoparticles proposed in the literature is given in Figure 5I (structures a to d)—so-called “Janus”, “brush”, “jellyfish”, and “pancake” conformations were taken into consideration as possible at the interface.^30−32^ However, published reports and visualizations concerned nanoparticles of a few nanometers in size and relatively long PNIPAM chains. In our case, the PNIPAM shell was thin compared to the diameter of the whole PSiFe NP. Therefore, we considered different aspects of the conformational changes of PNIPAM at the air/water interface.

First, the nanoparticle’s immersion was important (Figure 5II), as above the water surface, PNIPAM should be in “closed” conformation both at LT and HT due to the lack of hydration of the chains. The scenario in which particles were in the majority above the water surface (Figure 5II) was excluded. In such a case, neighboring particles always interact *via* “closed” PNIPAM irrespective of temperature. The area occupied by a single NP should not change significantly upon temperature change (neither the interactions nor aggregation). However, we observed clear shifts in the surface area occupied by PSiFe between LT and HT, which we correlated with the conformational changes of PNIPAM.

Therefore, we analyzed the interactions for immersed nanoparticles (50% or more). We restricted the discussion to spherical objects, neglecting the gravitational force.^39,77^ According to the literature, when the particle is small (*ca.* <5 μm), the partial immersion of a particle depends on the wetting properties.^39,60,78^ No meniscus (nor the capillary force) is present, and the water surface remains flat. The hydrophilic particles are more immersed in water than hydrophobic nanoparticles.

The observed change of area occupied by PSiFe at the interface in two temperature regimes should be at least around 8% but not more than 23%. The upper limit corresponds to PNIPAM not contributing to the overall size of the particles at HT and maximum elongation at LT. The approximation of *ca*. 8% was based on the geometrical model, where NPs were half-immersed, and bending of PNIPAM resulted in 35% shortening of polymer chains (*i.e*., maximal shortening according to Tucker and Stevens^46^). We compared area shifts of the isotherms, and the changes were around 10–20%, depending on the experimental run (Figure 2a). This proved that PNIPAM attached to the PSiFe nanoparticles performed an “open”-to-“closed” transition. “Closed” form was likely possible because of the interactions between neighboring PNIPAM oligomers at the surface of a single NP, as described by Shan *et al*.^47^

Interestingly, PSiFe particles were much larger than those previously studied by Shan *et al*.,^47^ but the impact of PNIPAM changes was still clearly visible. A capping layer of about 5 nm thickness controlled the properties of the particles with a diameter of 90 nm.

Besides the conformational changes from “open” to “closed”, interdigitation of the PNIPAM ligands attached to the neighboring particles could explain the shift of the isotherms. We analyzed the possibility that the polymer ligands became more flexible upon the increase of temperature, facilitating interdigitation at HT. First, the surface compressional moduli were slightly higher at LT (Figure 2a). This correlated well with the difference between PNIAPM in “open” *versus* “closed” conformation. “Open” conformation allowed for higher compressibility. By analyzing the interfacial dilatational elastic modulus and interfacial dilatational viscous modulus, we found that the elastic properties were dominant at both temperatures. The viscoelastic component should be more dominant for interdigitation, which was not observed here (Figure 2b). The relaxation experiments showed significant differences in PSiFe behavior at both temperature ranges. First, the relaxation time for LT was much longer than for HT, suggesting more interparticle interactions, which kept the particles together. Also, the analysis was performed using the Kohlrausch–Williams–Watts function. The value of β (0.62) at LT suggested strong cooperative motions, which was not noticed at HT. Additionally, PSiFe particles aggregated at LT and not at HT (BAM observations). Based on these results, we concluded that the “open”-to-“closed” transition of PNIPAM occurred, and the interdigitation of ligands could not explain the observed changes in the surface area between LT and HT.

These observations allowed us to describe the interactions occurring both upon compression and temperature change. Upon folding of PNIPAM, the hydrogen bonds were created. These could be intramolecular, intermolecular, and inter-nanoparticulate (intermolecular but between PNIPAM anchored to different PSiFe). Intramolecular hydrogen bondings were unlikely for such short PNIPAM chains (24 N). Inter-nanoparticulate bonding was excluded based on the lack of changes in interfacial dilatational elastic modulus and interfacial dilatational viscous modulus due to temperature change. Therefore, it was likely that hydrogen bonds were formed only between PNIPAM oligomers at the same nanoparticle in the studied system. Such an explanation was also reported for smaller nanoparticles.^47^ Hence, we reckoned that PSiFe NPs before compression at LT adapted the conformation visualized in Figure 5IIIa and during compression in Figure 5IIIb (and not Figure 5IIIc). No interdigitation could be caused by steric hindrances (side chains of PNIPAM) and the small curvature of relatively large nanoparticles.^79^

The surface potential measurements provided a piece of additional evidence for the investigation of the conformation of PSiFe. We analyzed the differences between values recorded at LT and HT (Figure 1c). We subtracted values for the given surface area recorded at HT from those measured at LT. In broad ranges of surface areas per nanoparticle, the surface potential at LT was higher than at HT. We rejected the possibility that these differences originated in more disordered capping layers (but without changes in conformation) at HT. Such an explanation required increasing differences between surface potentials at various temperatures with compression, as in the case of Nakahara *et al*.,^62^ which was not the case. Additionally, PNIPAM ligands were well fixed at the surface of PSiFe (*via* the silane group), and lateral rearrangement of the anchoring group was not possible.

This brought us to consider possible conformations more closely. PNIPAM was “closed” at HT in all regions of PSiFe, *i.e*., both under and above the interface. This resulted in no asymmetry and a smaller dipole moment. Above LCST, hairy nanoparticles adapted one of the following conformations: (a) “Janus” (Figure 5Ia), “brush” (Figure 5Ib), “jellyfish” (Figure 5Ic), or “pancake” conformation (Figure 5Ic). In the “Janus” conformation, PNIPAM chains above water were not hydrated and thus “closed”. In other conformations, the dipole moment’s vertical component was much smaller than submerged chains. Irrespective of the specific conformation adapted, PSiFe at LT showed a larger surface potential, dipole moment, and asymmetry.

The surface potential led us to a similar conclusion as Shan *et al*.^31^ provided for gold nanoparticles. The authors observed a blue shift of the SPR band with the compression. In their case, at LT, a few nanometer AuNPs were in the “pancake” conformation (Figure 5Ic). This locally exposed the immersed part of the NPs to water molecules by the horizontal arrangement of PNIPAM at the interface. The blue shift was observed simultaneously with the compression, and the rise of the surface potential was explained by the depletion of PNIPAM chains and covering the metal surface. This lowered the local polarity of the metal core proximity.^31^ PSiFe NPs were much larger than described AuNPs, so the deflection of polymer ligands was marginal because the capping layer of PSiFe was well fixed to the surface and short compared to the particle’s size. However, the change in dipole moment was registered, and we assigned it to the local polarity changes due to the presence of water at hydrated PNIPAM chains and folded PNIPAM with no water molecules nearby (LT). Considering the thermodynamical confinement of nanoparticles at the interface and the interpretation of the dipole moment, we concluded that the PSiFe nanoparticles adapted “Janus-like” conformation at LT (Figure 5II).

### Origins of Aggregation of the PSiFe Nanoparticles

Brewster angle microscopy revealed that PSiFe nanoparticles were more likely to aggregate at LT, showing fractal-like patterns. SEM also confirmed the different aggregation patterns at LT and HT (Figure 4). This was contrary to typical systems and hydrogels based on PNIPAM,^80^ where an increase in the temperature resulted in an opaque suspension of PNIPAM-stabilized particles. Films transferred at HT were uniform over large areas of at least around 4000 μm^2^. Films at LT were not uniform, distorted, and areas of varying local concentrations of NPs were clearly visible (Figures S8 and 4b). SEM pictures also revealed that the same surface pressure corresponded to a larger interfacial area per NP at LT. This was caused by the open conformation of PNIPAM or a “cloud” of water molecules that hydrated the PNIPAM.

As the analysis of the conformation change of PNIPAM at the surface of PSiFe and experimental data allowed us to exclude interdigitation, we considered other possible origins of the behavior of PSiFe at the air/water interface and the solid substrates. Bresme *et al*.^77^ listed five main interactions to be considered in liquid surface-deposited nanoparticles: capillary, electrostatic, van der Waals, fluctuation (thermodynamic Casimir force), and solvation interactions.

The important result in explaining the PSiFe behavior was the formation of dendrimer-like structures at LT observed under BAM (Figure 3g). Reynaert *et al*.^81^ studied sulfonated polystyrene microparticles (3.1 ± 0.2 μm in diameter) at the decan–water interface. They showed that fractal aggregation was induced by dissolving NaCl in the subphase. The aggregation was faster for the higher salt concentration. NaCl was used to weaken the electrostatic repulsion of the particles. In our case, the fractal aggregates were present at LT, suggesting weaker repulsion. Alternatively, the short-range attraction might be weaker at HT.

The significance of lateral capillary forces also explains similar observations.^81,82^ Because capillary interaction depends on the local curvature, an additional particle is most likely to attach to an aggregate at a point where the curvature has the largest gradient.^81^ This is recognized as diffusion-limited cluster aggregation.^81^ However, lateral capillary forces should not be present in our case due to the size of the particles.^39^

Fluctuation force was reported as the fractal aggregates genesis by Boniello *et al*.^83^ They studied the behavior of partially wetted micrometer-sized particles at an air/water interface. The investigated particles diffused faster when they were more immersed in water. That was counterintuitive in terms of the viscosity of the fluids^83^ and was explained through the thermal fluctuations of the interface.

The main conclusion on interaction was that temperature-induced aggregation in the PSiFe system caused the change of the attraction force-to-repulsive force ratio. The fractal-like pattern formation was governed by diffusion-limited aggregation or diffusion-limited cluster aggregation. The true origin of those antagonistic factors and methods to precisely control them is the next step in advanced nanoengineering.

## Conclusions

Switching/responsive domains are still dominant in both molecular structure and complexity in most adaptive systems. Here, we show that even relatively short ligands (around 5 nm) can be effectively used to control the behavior of large particles (approximately 90 nm in diameter). This might be crucial for incorporating various domains, *e.g*., providing responsiveness to external stimuli (light, temperature, pH magnetic field, *etc*.) or performing specific actions (catalytic, transport, sensing, targeted binding, *etc*.), into single dynamic systems.

The current research stage presents PSiFe nanoparticles as a promising building block in creating complex self-assembly systems. Recently, an exciting application of similar PNIPAM-capped SiO_2_ NPs was shown.^29^ The authors presented a liquid–solid switchable macroscopic device. Their study describes that approximately 200 nm in size nanoparticles with around 8 nm of the capping layer were able to create inter-nanoparticulate hydrogen bonds in dense suspension due to temperature change. We did not observe such transition within the interfacial system composed of similar particles (PSiFe, approximately 90 nm in diameter with around 5 nm of capping layer). Slightly shorter PNIPAM ligands allowed only for inter- or intraligand hydrogen bonding within a single nanoparticle but not for the interdigitation of neighboring capping shells.

Langmuir films of PSiFe could switch between two states of films: uniform and distorted. At HT, uniform films over large areas were formed, and aggregation was barely visible. Below the LCST, films tended to form fractal, dendrimer-like agglomerates capable of disaggregation only at low surface pressure. In typical systems based on PNIPAM, the aggregation occurs above the LCST. The switching of the properties also manifested in the recorded isotherms. Higher surface pressure was observed for the “open” (below the LCST) and “closed” nanoparticles (above the LCST) at the same compression rate and the same amount of the PSiFe. The current analysis reveals that the binary states of the systems come from a mix of long-range repulsion and short-range attraction. The balance between them is interrupted by the temperature impacting diffusion-limited aggregation. This feature will be useful for engineering interfacial switchable membranes. BAM revealed that the optical properties of the monolayer were tunable due to the compression and the temperature. An AuNP-based window-mirror, which was based on a similar phenomenon, was recently constructed.^84^

## Supporting Information Available

Additional SEM pictures of Fe*_x_*O*_y_*@SiO_2_ and PSiFe, DLS size distribution of Fe*_x_*O*_y_*@SiO_2_ and PSiFe, ζ-potential measurements at LT and HT, details of the stability experiments (including the graphs normalized to the area/mass, surface potential, additional explanations related to elasticity and viscosity measurements, calculations regarding trapping energy), and description of magnetic properties of PSiFe (PDF)

## Abbreviations

PSiFe: Fe*_x_*O*_y_*@SiO_2_ nanoparticles with poly(*N*-isopropyl acrylamide) (PNIPAM) grafted on the silica surface
NP/NPs: nanoparticle/nanoparticles
SPR: surface plasmon resonance
HT: high/higher temperature, referring to 40–42 °C
LT: low/lower temperature, referring to 18–20 °C
