# Supplementary material for: Stimuli-Responsive Langmuir Films Composed of Nanoparticles Decorated with Poly(N-isopropyl acrylamide) (PNIPAM) at the Air/Water Interface
Source: ACS Omega. 2023 May 31;8(26):23706–19. doi: 10.1021/acsomega.3c01862 (PMC10323952; doi:10.1021/acsomega.3c01862)
Supplement: Supplementary file 1 — ao3c01862_si_001.pdf [file ao3c01862_si_001.pdf]

Supporting Information

Stimuli-responsive Langmuir Films Composed of Nanoparticles Decorated with Poly(N-isopropyl acrylamide) (PNIPAM) at the Air/Water Interface

*Rafał Zbonikowski, Michalina Iwan, Jan Paczesny**

Institute of Physical Chemistry, Polish Academy of Sciences, Kasprzaka 44/52, 01-224 Warsaw, Poland E-mail: jpaczesny@ichf.edu.pl

Characterization of the nanoparticles

$Fe_xO_y@SiO_2$ nanoparticles

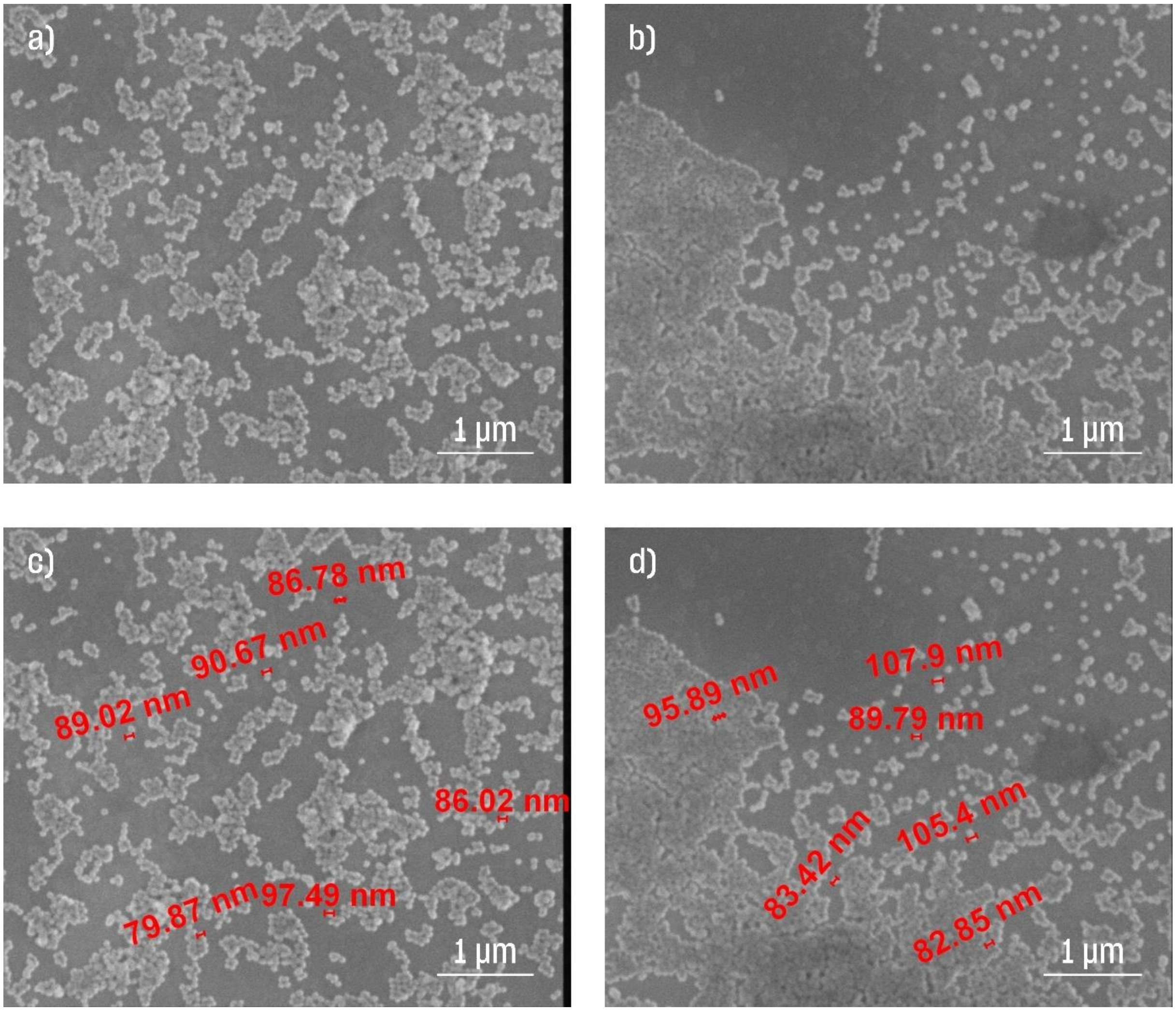

Figure S1. SEM pictures of $Fe_xO_y@SiO_2$ nanoparticles (a-d) (without PNIPAM ligands).

			Size (d.nm):	% Volume:	St Dev (d.nm):
Z-Average (d.nm):	93,15	Peak 1:	85,99	100,0	24,71
PdI:	0,082	Peak 2:	0,000	0,0	0,000
Intercept:	0,922	Peak 3:	0,000	0,0	0,000
Result quality :	Good				

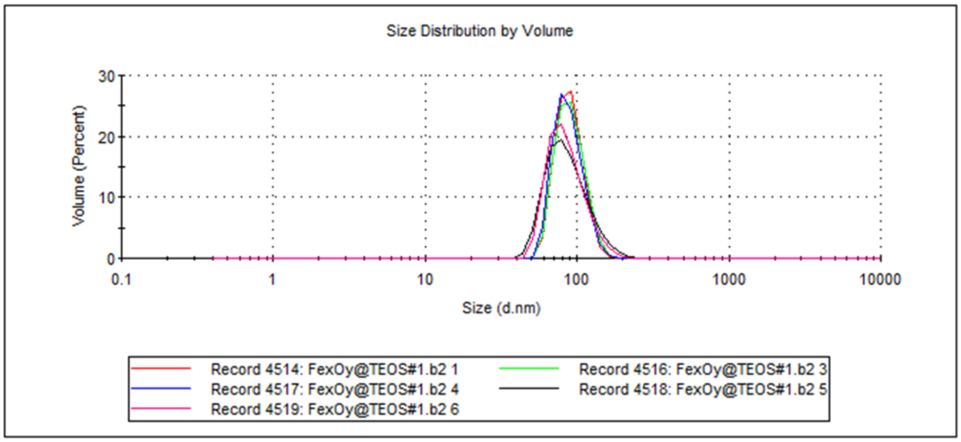

Figure S2. Size distribution of $Fe_xO_y@SiO_2$ nanoparticles (DLS) (without PNIPAM ligands).

PSiFe ($Fe_xO_y@SiO_2@PNIPAM$) nanoparticles

			Size (d.nm):	% Volume:	St Dev (d.nm):
Z-Average (d.nm):	130,1	Peak 1:	114,2	95,5	56,03
PdI:	0,216	Peak 2:	5224	4,5	754,1
Intercept:	0,919	Peak 3:	0,000	0,0	0,000
Result quality :	Good				

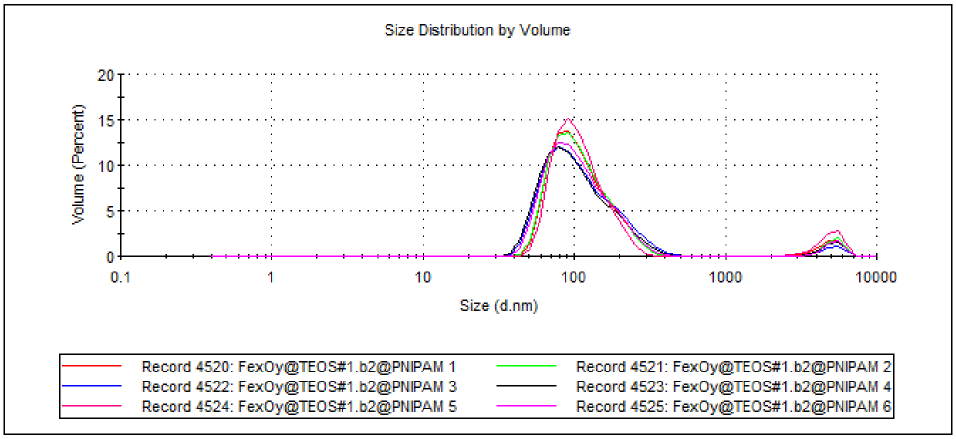

Figure S3. Size distribution of PSiFe nanoparticles (DLS).

The analysis of DLS measurements of the diluted sample (1 mg·ml^{-1}) revealed that the average size (by volume) was similar at both experimental temperatures (100 nm and 90 nm at 40 °C and 20 °C, respectively). However, the standard deviation of the sample at 40 °C was higher (35 nm *vs*. 31 nm). The expected change in the size of PSiFe due to changes in the conformation of PNIPAM was in the range from around 10% to around 20%, and it was not visible in DLS measurements, most likely due to the polydispersity of particles. SEM confirmed the polydispersity of the cores (**Figure S1**).

We do not treat those average sizes as differentiating compared to the other results and experiments. Our analysis of Langmuir isotherms proved a change in the size of PSiFe upon temperature changes. Also, the average distances between the surfaces of cores were much smaller for **HT** compared to **LT** (*cf*. **Figure S11**). However, when we analyzed the distances between the centers of the cores, we did not observe any significant differences between both temperatures due to the polydispersity of the cores.

The decrease of surface zeta potential (**Table S1**) due to the temperature change matched the theory of the stability of PSiFe nanoparticles. Stable colloid below LCST became less stable above the LCST of PNIPAM. In "closed" conformation, PNIPAM became more hydrophobic, thus having a smaller absolute value of zeta potential at **HT** (around -42 mV) than at **LT** (around -55 mV).

Table S1. Zeta potential measurements of PSiFe nanoparticles.

Zeta Potential			
20 °C		40 °C	
Zeta Potential	Standard Deviation	Zeta Potential	Standard Deviation
-54.62 mV	13.10 mV	-41.87 mV	15.77 mV

Supplementary results

The stability of PSiFe films at the interface

Data for three consecutive compression/decompression cycles without changing the temperature regimes in between cycles (as complementary to **Figure 1 d)** in the main text) is shown in **Figure S4**. Isotherms at **HT** were reproducible (left). Cycles at **LT** (right) were poorly reproducible, which reflected the tendency of PSiFe to aggregate in this temperature regime.

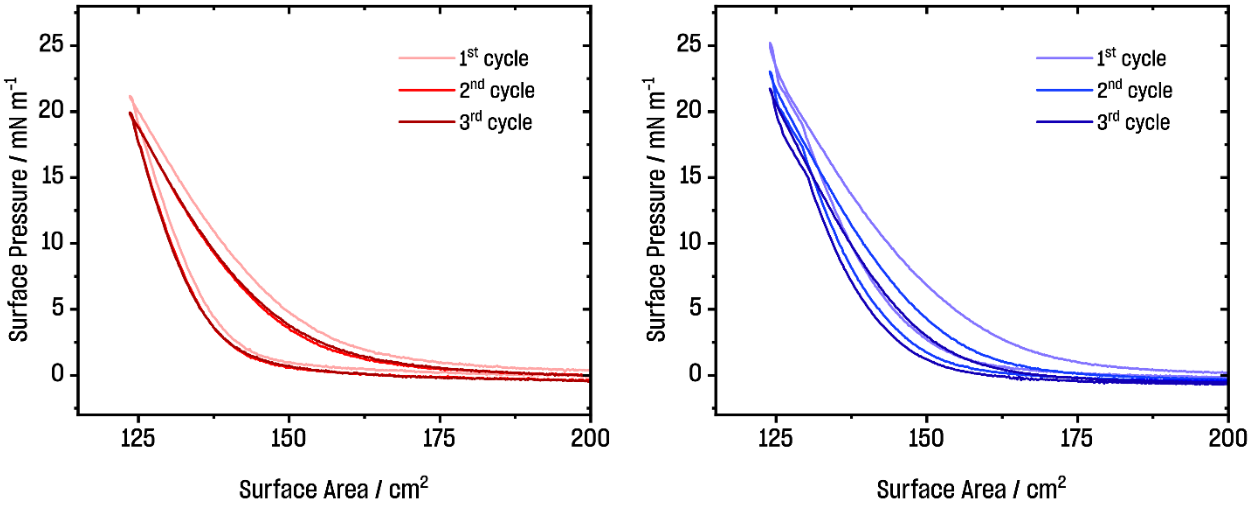

Figure S4. Three consecutive compression-decompression cycles of PSiFe films at **HT** (left) and **LT** (right). Isotherms at **HT** were reproducible. However, at **LT**, some irreversible aggregation occurred.

We judiciously chose 7.5 mN·m^{-1} to show the linear relationship between the deposited volume of PSiFe suspension and surface area (**Figure 1**). We did not follow the standard estimation of contact cross-sectional area (CCSA). This was done because the surface pressure increase started early but with a small slope. Even minuscule changes in inputs resulted in a considerable variation in CSSA. Analogical calculations were conducted for 0.5 mN·m^{-1}, 1.0 mN·m^{-1}, 2.5 mN·m^{-1}, and 5.0 mN·m^{-1} leading to the same conclusion.

The compression isotherms were recalculated to unify the amount of sample deposited on the interface (**Figure S5 a) b)**). The three isotherms for both temperatures were close to each other. However, adding consecutive portions of PSiFe resulted in a relatively small shift towards smaller surface area values. These deviations arose from consecutive compression/decompression cycles. Upon compression, a small number of particles could drown into the subphase, or aggregates could be formed, which did not entirely spread upon decompression.

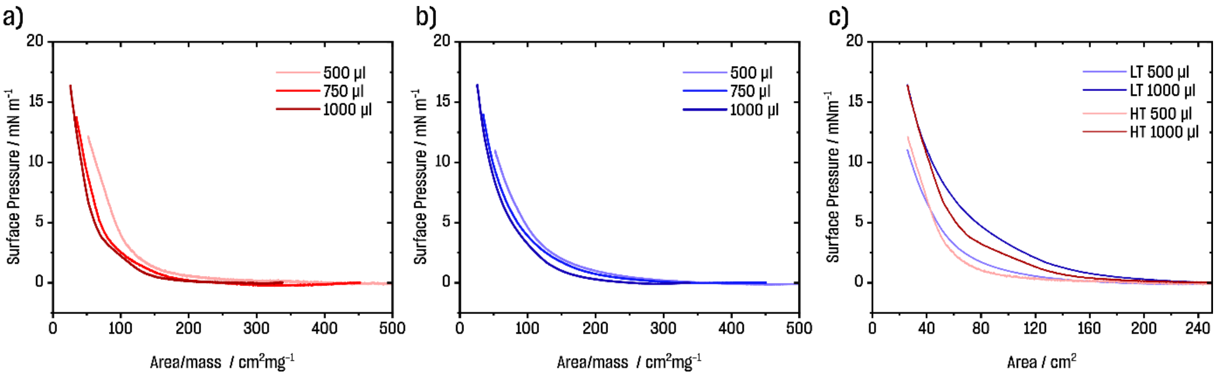

Figure S5. **a)** Stability experiments of the PSiFe nanoparticles on the air-water interface, 40 °C, normalized to the area/mass; **b)** Stability experiments of the PSiFe nanoparticles on the air-water interface, 20 °C, normalized to the area/mass; **c)** comparison of isotherms from **Figure 1 a)** and **b).**

Surface potential and dipole moment

Surface potential was measured simultaneously with the surface pressure. The potential rises slowly and almost evenly at the **HT** with the surface pressure (**Figure S6 a)**). Close to the maximal compression of the film, a rapid increase in the surface potential was observed. This might be related to the artifact caused by the proximity of the barriers (having paramagnetic parts) to the Kelvin electrode. However, this sudden jump in surface potential was not visible at **LT** (**Figure S6 b)**).

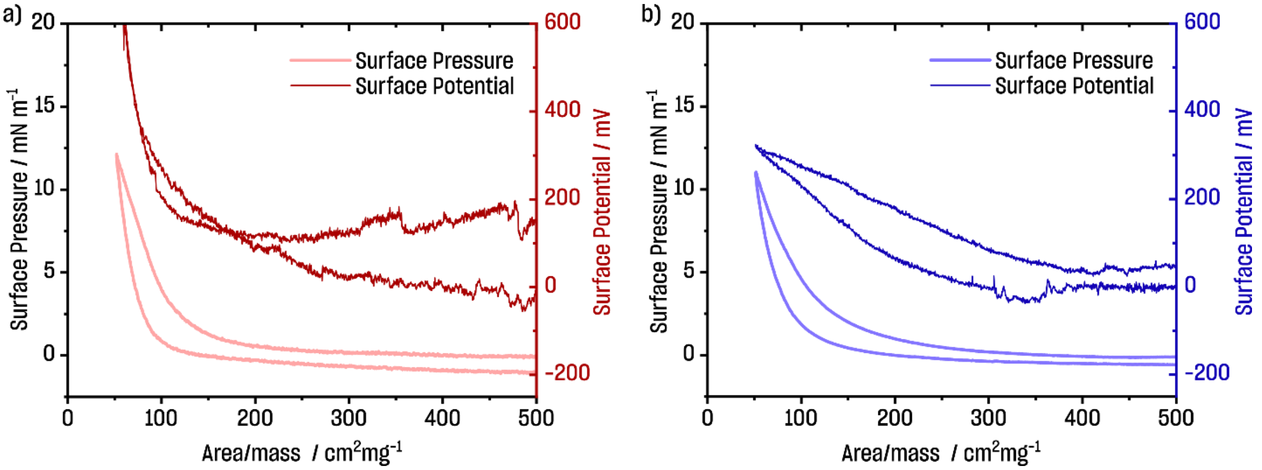

Figure S6. The surface potential (vivid colors) at high (**a**) and low-temperature regimes (**b**) and surface pressure isotherms of those compression-decompression cycles (pale colors).

We calculated the surface potential and dipole moment differences between temperature regimes based on the experimental data presented in **Figure S6**. These differences are shown in **Figure 1 d)** (main text). The area per nanoparticle was fixed according to the size of the nanoparticles, and the close-packed hexagonal structure of cross-sections was assumed. The dielectric permittivity (ε) of the layer changed linearly from 1 to 3.9 (the value for SiO_2), consistent with the area per nanoparticle change. That was used to compensate for the unknown parameter of the monolayer permittivity, which changed in time upon the compression. The calculations were carried out to illustrate the dipole moment's difference. The positive value indicated that the dipole moment (perpendicular to the interface) of PSiFe was higher at **LT**.

Rheological measurements

The monotonic increase of surface compressional modulus was observed upon compression at both temperature regimes. The inflection point was not reached, suggesting we operated below the

collapse. This was important to explore the dynamic properties of the films without the risk of irreversible aggregation.

Fully “closed“ conformation of PNIPAM (at **HT**) resulted in lower values of surface compressional modulus compared to “open” ligands (at **LT**) during the whole compression. This was fully in line with the expectations. At the lower temperature, particles interacted *via* “open” PNIPAM chains (**Figure 5 III a)**). “Closed” conformation restricted the possible interactions between particles compared to “open” conformation.

During the oscillatory barrier method experiments, a slight drift in surface pressure values was observed. Hilles et al.[1] discussed several possibilities for the drift to occur. First, instead of being caused by a plastic action, the decrease in background pressure could result from chain desorption from the interface followed by dissolution in the subphase. Also Morioka and Kawaguchi[2] discussed such a possibility. The other explanation by Hilles et al.[1] was that the stress caused in the xy-plane was compensated by deformation along the z-coordinate. The polymers elongated in the direction perpendicular to the interface. We pointed out two crucial factors in PSiFe experiments. At **LT**, the drift originated in a very long relaxation of the films (*cf. Rheological measurements: relaxation experiments*), as shown in the main text in **Figure 2 c)** (around 54 min for **LT**). We assumed that allowing the system to equilibrate longer (60 min) was enough, but it was visible in the Lissajous diagrams that the small drift was still present. At **HT**, it was most likely related to the evaporation of water at a relatively higher temperature. Small changes in water level reflected in the Lissajous diagrams. In other words, there was a background, and the depreciation discussed by Hilles et al.[1] and Morioka and Kawaguchi[2] was not intrinsic to the system.

We performed analysis according to Hilles et al.[1] to prove that the oscillatory barrier method experiments were performed in the linear regime. In **Figure S7**, we plotted the surface pressure as a function of changes in moduli of area (to reflect compression and decompression). The upper row shows single compression at both **LT** and **HT** and a frequency of 0.10 Hz. The bottom row show multiple full cycles but with the drift corrected. Separated, individual cycles were linear without the correction.

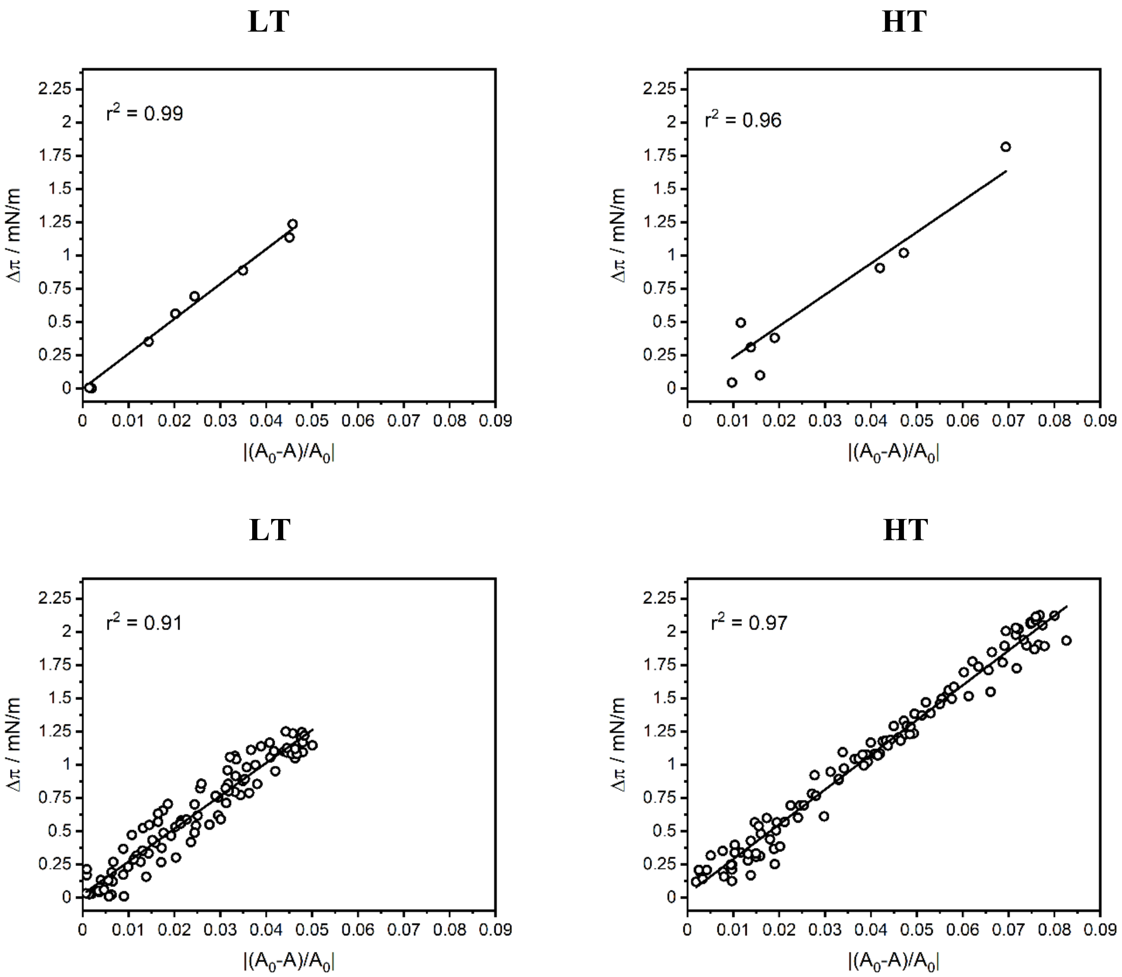

Figure S7. The changes in surface pressure as a function of moduli of area changes during the oscillatory barrier method experiment at the frequency of 0.10 Hz. The upper row shows single

compression. The bottom row show multiple full cycles but with the drift corrected. No significant deviation from linearity was observed.

Additionally, we followed the protocol proposed by Guzman to verify the total harmonic distortion (THD) parameter[3]. THD measures the amount of harmonic distortion present in a signal. First, we performed Fourier transformation to identify fundamental and harmonic frequencies in the surface pressure readout upon oscillatory barrier method (0.10 Hz, both **LT**, and **HT**). The results are presented in **Figure S8**. Next, THD was calculated as $\sqrt{\frac{\sum harmonic\ amplitudes^2}{funudamental\ amplitude^2}}$. THD values were around 0.07 at **LT** and 0.12 at **HT**. These values were similar to THD calculated by Guzman et al. for 1,2-dipalmitoyl-sn-glycerol-3-phosphocholine (DPPC) upon oscillation with amplitudes of the area change in the range from around 5% to 10%[4].

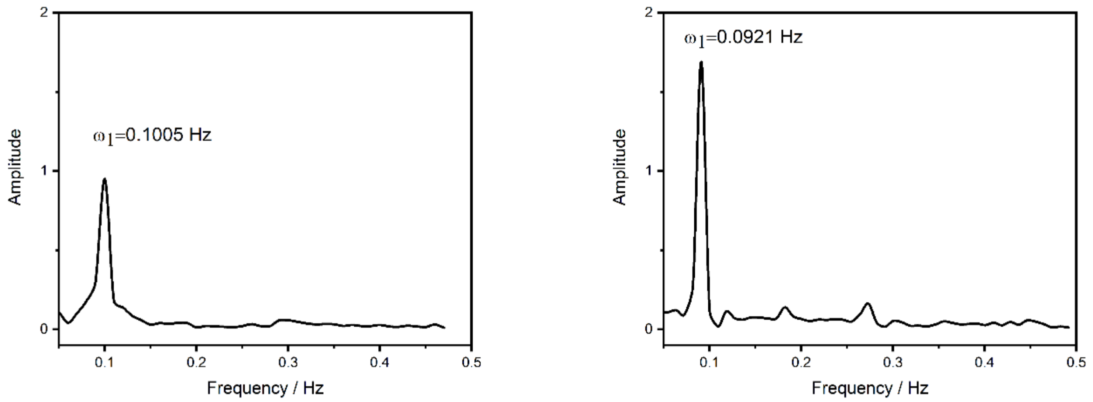

Figure S8. FFT spectra of the surface pressure signals were recorded during the execution of oscillatory barrier method experiments at **LT** (left) and **HT** (right) with a frequency of 0.1 Hz.

Additional Lissajous diagrams (0.05 Hz) are shown in **Figure S9**.

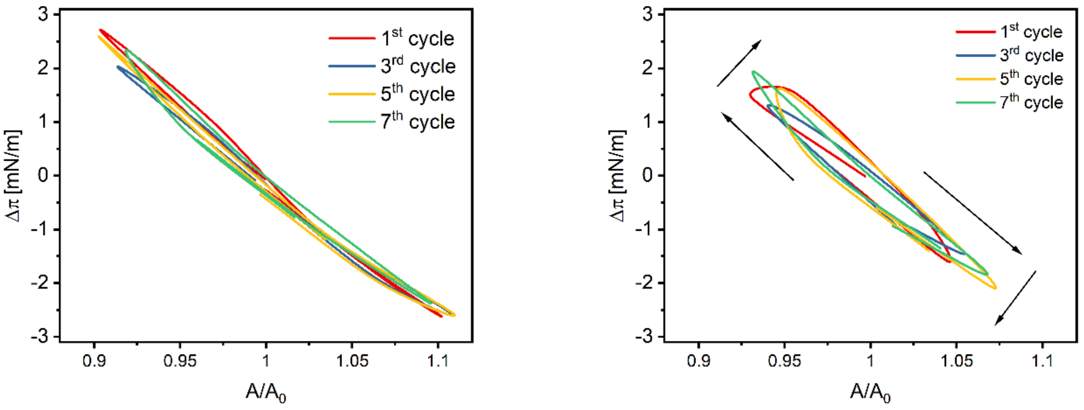

Figure S9. Lissajous diagrams for PSiFe films (0.05 Hz) at **HT** (left) and **LT** (right).

The trapping energy

We assumed that PSiFe particles were small enough, so they were irreversibly trapped at the interface. Here, we provide both theoretical and experimental explanations for such an assumption.

Theoretical explanation

The energy of entrapment of a particle at the interface can be expressed with the following formulas [5,6]:

$$E = -\frac{\pi R^2}{\gamma_{1,2}}(\gamma_{1,2} - (\gamma_{P1} - \gamma_{P2}))^2$$

For spherical particles (minus within the brackets describes desorption into the water, while plus – removal of the particle into air[7]:

$$E = -\pi R^2 \gamma_{1,2}(1 \pm \cos\theta)^2$$

where R – radius of a particle, $\gamma_{1,2}$ - surface tension of air/water interface, θ – contact angle, $\gamma_{P1,P2}$- surface tensions of solid particle and a fluid phase (water or air), surface tensions of air-water

interface in 20 °C and 40 °C. $\gamma_{1,2} = 72.75$ mN·m^{-1} for 20 °C and $\gamma_{1,2} = 69.60$ mN·m^{-1} for 40 °C, R = 45 nm [8].

Table S2. Comparison of kT and energies needed to remove a particle from the interface at 20 °C and 40 °C

	**20 °C**	**40 °C**
**kT**	4.04737·10^{-21} J	4.32350·10^{-21} J
**Range of energies needed to remove a particle from the interface**	0-1.85126·10^{-15} J	0-1.77110·10^{-15} J

Contact angles, at which kT is enough to remove a 90 nm-particle from the interface into the water, were calculated: for 20 °C θ = 4.41° and for 40 °C θ = 4.53°. The contact angle of PNIPAM microgel varies between 30-50° below LCST to 80-90° above LCST[9]. For θ = 30° and T = 20 °C, the energy needed to remove a particle into water is approximately 2000 kT, and for θ = 80° and T = 40 °C the energy is approximately 73000 kT.

Zang *et al*. presented a complex study on the influence of the contact angle of silica nanoparticles at the air/water interface and the mechanical properties of the layers composed of these particles[10]. They used silica particles of 20 nm in diameter, with a hydrodynamic radius in a range from 72 nm to 87 nm. The contact angle of their nanoparticles changed from around 130° to around 20° with the increasing percentage of SiOH. The authors analyzed percentages from 20% to 100% and

presented stable films. The highest maximum compression moduli were obtained for 34% and 51% of SiOH (above 100 $mN \cdot m^{-1}$).

Applying the same calculations to their system, the absolute values of the entrapment energy at 20 °C were approximately equal to 19 kT for 20° and 675 kT for 130°. 20° and 130° are extreme contact angles tested by the authors, while the optimum contact angle leading to the highest adsorption energy was equal to 90°[10].

In this method, other capillary forces are neglected. For particles smaller than 5-10 μm with Eötvös number not much less than 1, the deformations of the interface due to the gravity are negligible as well as lateral flotation[5,11].

Experimental explanation

Consecutive addition of deposited sample caused an almost linear shift of the isotherms (**Figure 1 a), b)** and **Figure S4 a), b)**). At this point, we could not exclude the possibility of desorption because the b parameter (y=ax+b) of linear regressions for 0.5 $mN \cdot m^{-1}$, 1.0 $mN \cdot m^{-1}$, 2.5 $mN \cdot m^{-1}$, and 5.0 $mN \cdot m^{-1}$ was not equal to 0. However, the linear characteristics of surface pressure meant that if desorption from the interface occurred, it was always the same percentage of deposited nanoparticles. A much more probable explanation of the observed phenomena was that we deposited a partially pre-aggregated sample or sample partially aggregated during deposition. Another explanation was that the proposed method of deposition (using a glass rod instead of direct deposition of droplets onto the interface) had a certain efficiency. The efficiency might be lower when some part of the sample drowned due to gravitational flowage on the glass rod or pre-aggregates were created. Those phenomena explained the linear character of

consecutive deposition with the b parameter not equal to 0. In other words, the linear character meant that the nanoparticles which were entrapped on the interface should not desorb due to kT.

SEM picture analysis

Figure S10 presents a larger area of PSiFe Langmuir-Blodgett film at **LT**. Pictures shown in the main text represent the films from a closer view. The whole film at **HT** was closely assembled as in **Figure 4 a)**, while the **LT** picture (**Figure 4 b)**) had both regions of close packing and free spaces between nanoparticles.

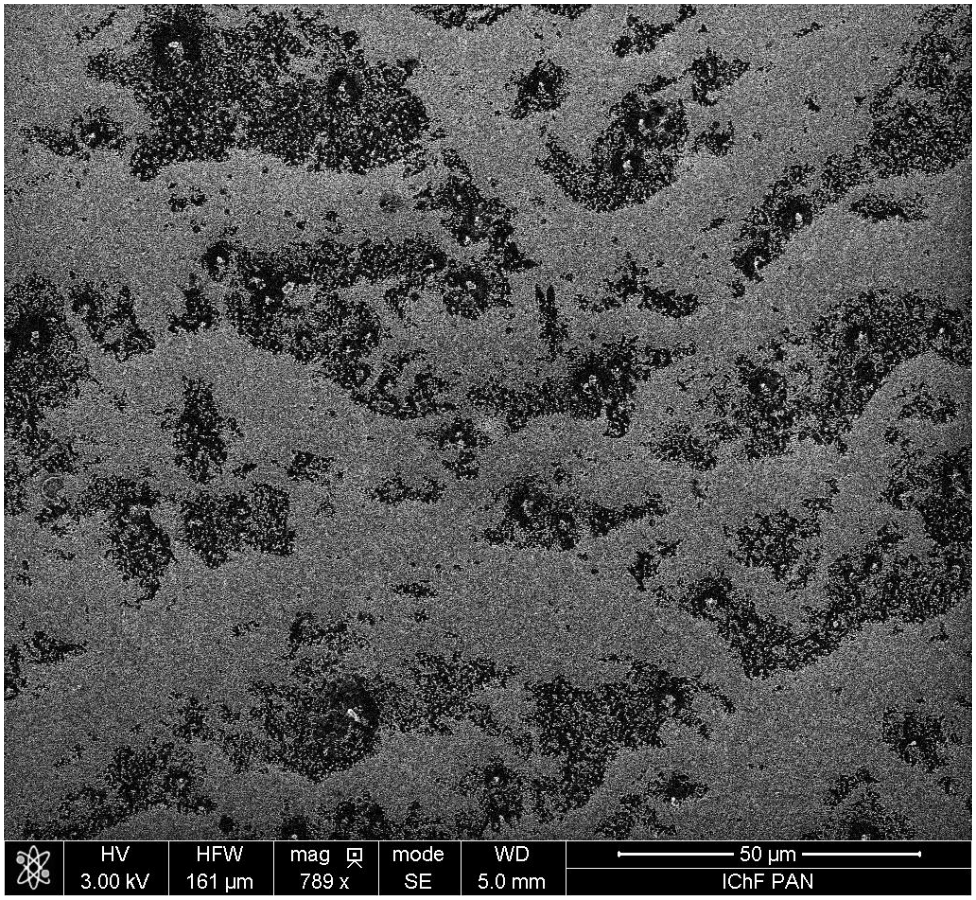

Figure S10. Large area PSiFe NPs distribution within Langmuir-Blodgett film transferred onto a silicon wafer at 20 °C (**LT**).

The origin of the temperature-induced shifting of the isotherms was the change in the ligand structure from "open" at **LT** to "closed" at **HT**. Additional analysis of SEM pictures clearly showed that the particles at **LT** were further apart compared to **HT** (**Figure S11**). The analysis was done using ImageJ, and the distance between closest neighbors was measured. All particles further away than 30 nm were neglected.

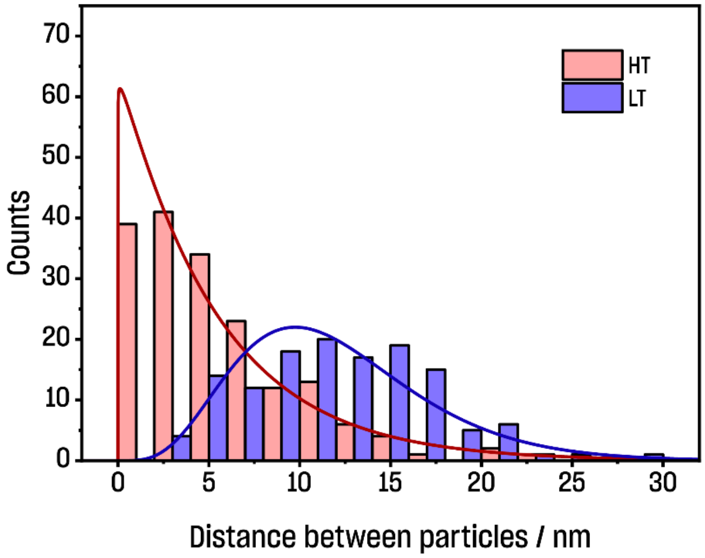

Figure S11. Histogram showing the distance between cores of PSiFe at **HT** and **LT**. Particles were located closer to each other at **HT** than at **LT** because of PNIPAM being "closed" (above LCST) and "open" (below LCST), respectively.

We also performed additional SEM imaging of Langmuir-Blodgett films transferred at **HT** (**Figure S12**) and **LT** (**Figure S13**), both at low (5 $mN \cdot m^{-1}$) and high (25 $mN \cdot m^{-1}$) surface pressures. PSiFe particles were dispersed over the area at low surface pressure and **HT** (see inset in **Figure S11**). At low surface pressure, most PSiFe formed large islands at **LT**, which corresponded to condensed regions recorded in **Figure S10**. At high surface pressure, films at **HT**

were more uniform than **LT** (which was in line with observations at 9 mN·m^{-1}). Both at **HT** and **LT,** multilayer formation was visible but without any sign of collapse at the surface pressure isotherm curves (cf. **Figure 1 d)**) in the main text). We assumed that transitioning from 2D to 3D was not a sharp event but rather a continuous process that started above some threshold value (larger than around 10 mN·m^{-1} and lower than 25 mN·m^{-1}).

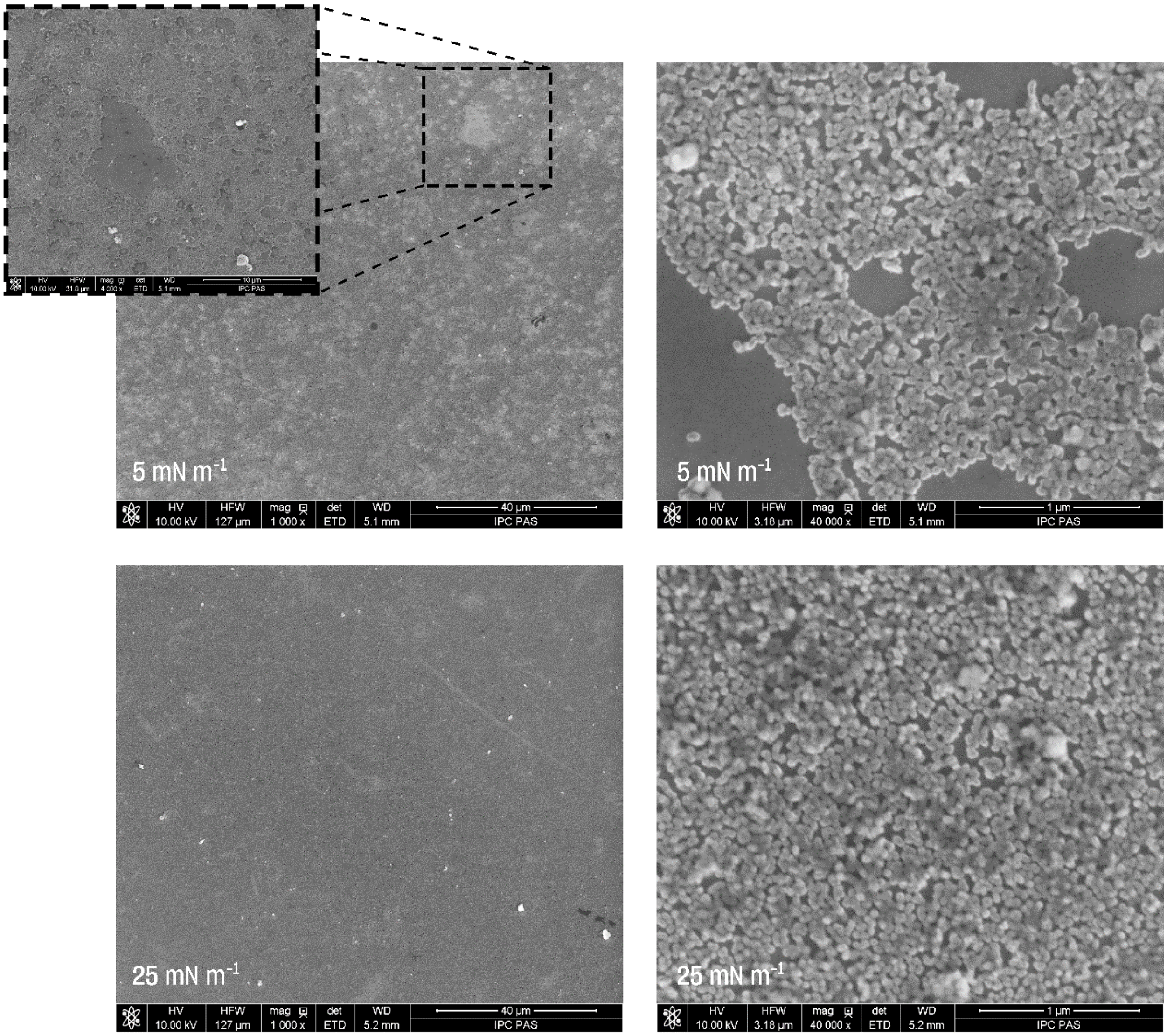

Figure S12. SEM pictures of films transferred onto silica wafers at **HT** at 5 mN·m^{-1} and 25 mN·m^{-1}.

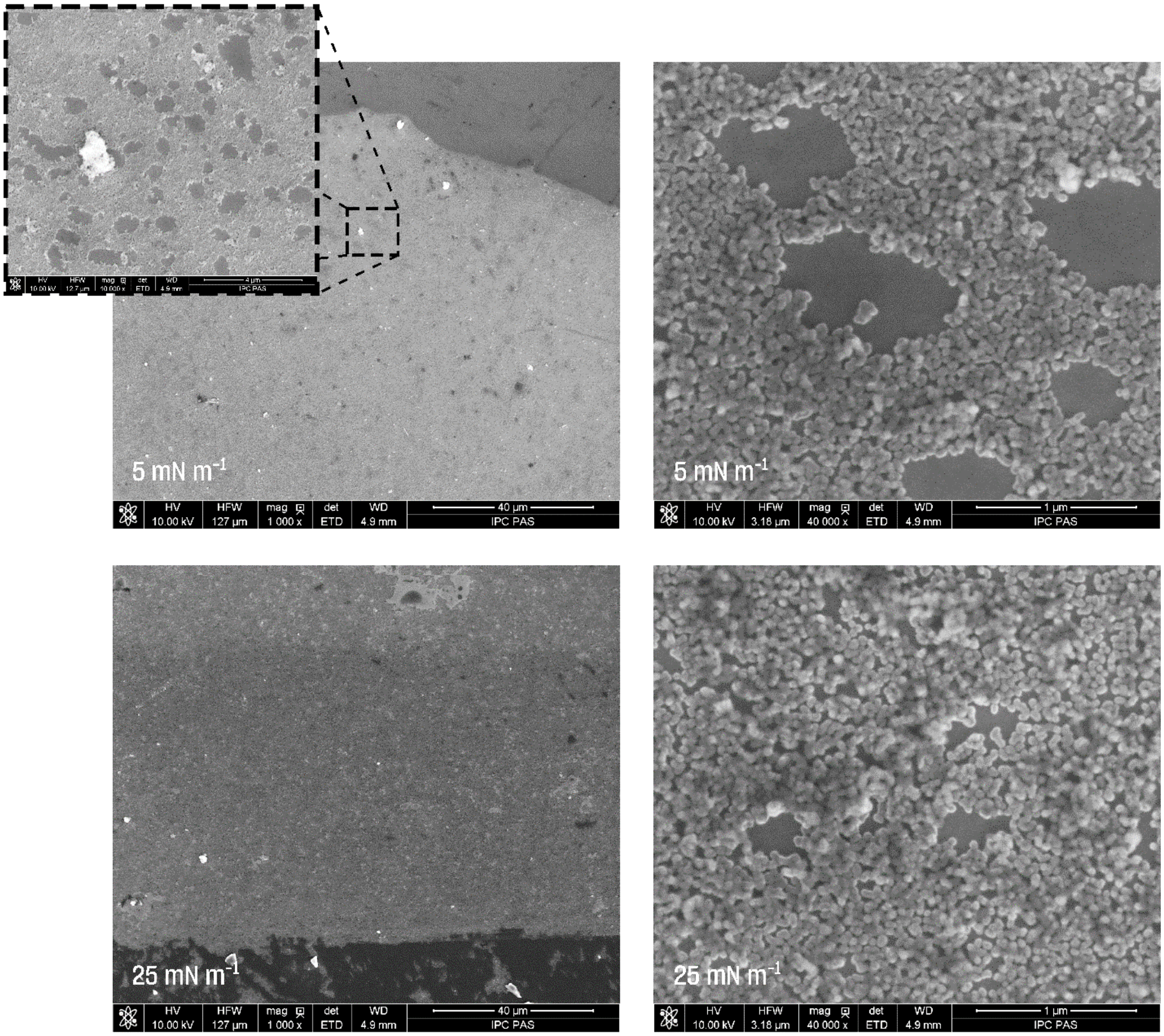

Figure S13. SEM pictures of films transferred onto silica wafers at **LT** at 5 mN·m^{-1} and 25 mN·m^{-1}.

Magnetic properties

The NIMA Technology set was used to transfer the monolayer with or without the neodymium magnet. The silicon wafer was placed in the dipper and partially immersed in the subphase. Depending on the variant, the neodymium magnet was partially submerged next to it, providing the constant and strong magnetic field (approx.. 0.17 T for **LT** and 0.12 T for **HT** in the proximity of the Si substrate) during the Langmuir monolayer creation and its transfer onto the Si substrate.

PSiFe nanoparticles (500 µl) were deposited on the water surface at 40 °C using the glass rod and the Hamilton syringe. Then, the system was cooled down to the temperature of 20 °C. The process took around 2 h. The monolayers were transferred onto the Si plates at the maximal possible area. The used amount of the sample, as estimated, should allow the free organization of the nanoparticles in the magnetic field.

A droplet of regular sample PSiFe nanoparticles was deposited onto a cleaned silica plate located in an open plastic chamber. Two neodymium magnets surrounded the chamber, providing the constant parallel lines of the magnetic field (approx. 0.44 T). After the evaporation of the solvent, the magnets were removed, disturbing the magnetic field. An analogical experiment without magnets was carried out as a control. Such prepared films were further investigated by SEM.

The results were not consistent nor reproducible. In the number of attempts, we observed anisotropically orientated domains (**Figure S14**).

It is crucial to underline that the precipitated PSiFe (*i.e.*, after a month of storage) were responsive to the same magnets, and their movement was undeniably visible (the precipitate color was brownish).

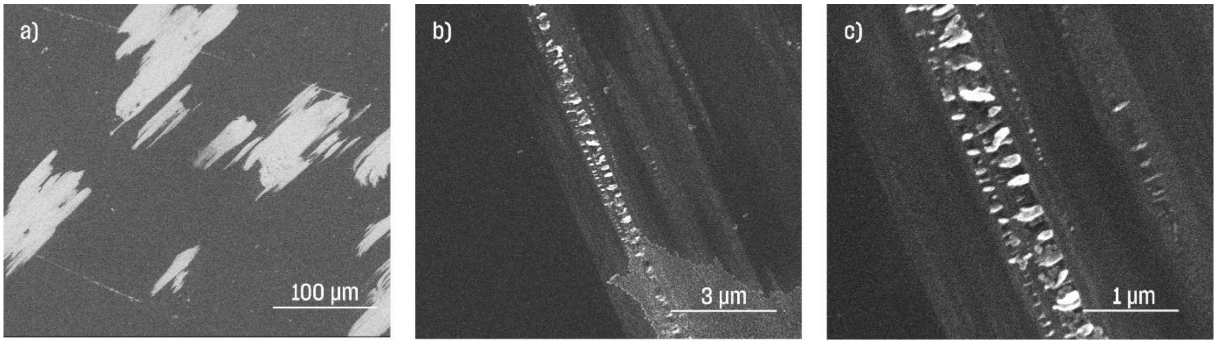

Figure S14. The magnetic domains of PSiFe were observed during the evaporation of the nanoparticles' dispersion in the magnetic field or Langmuir-Blodgett films transferred in the external magnetic field. **a)** lower magnitude; **b)** and **c)** higher magnitude.

Isotherms of non-thermo-responsive materials

Films of non-thermoresponsive particles or amphiphiles compressed to the same surface pressure but at a higher temperature should have a larger surface area due to thermal expansion. That is, the isotherm would be shifted to the right. Suppose a plateau or inflection on the isotherm indicates a phase transformation. In that case, Clapeyron's equation for phase transformations in 2D shows that if (dp/dT) is positive (only for water, ammonia, and a few metals is negative), then with increasing temperature, the pressure at which the transformation occurs is higher. The collapse occurs at lower surface pressure if the phase transformation is not there. The film collapses more readily at higher T due to thermal fluctuations. This was observed in the literature before[12].

We performed a control experiment by compressing stearic acid at **LT** and **HT** (**Figure S15**). The results matched the theory. Therefore, our observation of PSiFe isotherms shifting toward the left (lower surface area values) could not be related to the thermal agitation of the particles. Such shift (consistent throughout various experiments, including rheology experiments) was associated with the conformational changes within PSiFe.

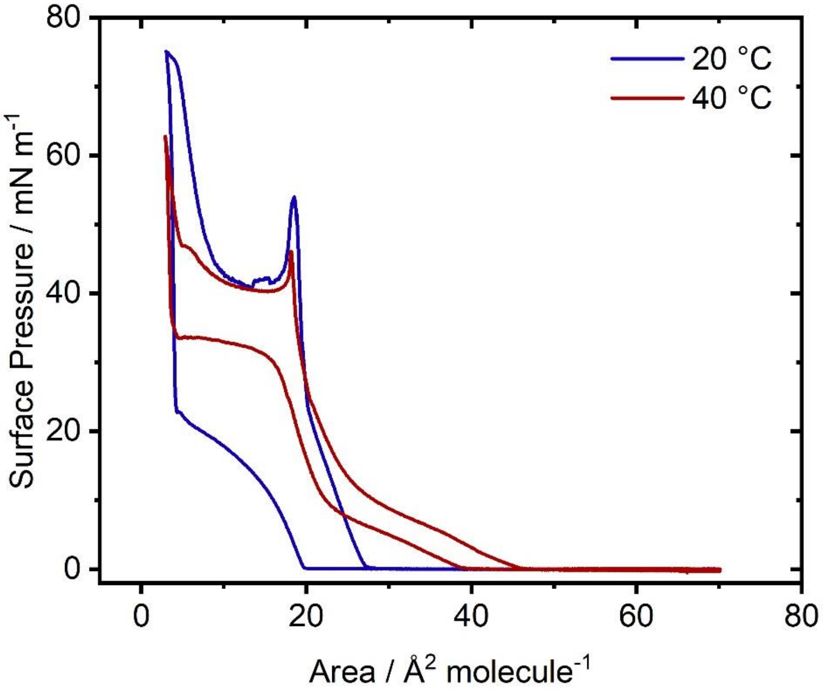

Figure S15. Control experiment showing compression of stearic acid at **LT** and **HT**. The opposite behavior to PSiFe was observed (as expected): *i)* there was no shift of isotherm at higher surface pressure values and close to collapse. In this regime, the size of the molecule, which did not change with temperature, was the limitation; *ii)* at lower surface pressure values, the isotherm was shifted to the right at the higher temperature due to thermal expansion.

The temperature gradient in the system does not cause instabilities

We performed computer simulation using the finite element method by employing Comsol software. We found that in the system, the temperature gradient was minimal (the largest we observed was 0.1 K between the bottom and the top of the trough). This was because it was not only the water heated up but also the air in the proximity of the surface. With such a small temperature gradient, we neglected the impact of convection on the observed phenomena.

We simulated a PTFE block with 5 mm-thick walls and a 1 mm-thick bottom and heated/cooled from the bottom (40 °C/20 °C). The ambient temperature was set to 23 °C. We put the container into the large enclosure filled with air (**Figure S16**, right upper panels). The system was equilibrated for 60 minutes (blue line) and 90 minutes (green line).

The most pronounced gradients were found when the trough was fully filled with water, *i.e.*, the depth was 9 mm (**Figure S16**, left, upper panel), and the temperature was set to 40 °C. The gradients depicted in the bottom panels of **Figure S16** were calculated along the red lines in the top left panel. The bottom left panel shows the temperature gradient from the heating element to the surface of the water, whereas the bottom right panel shows the gradient only in water. The differences in water temperature reached only around 0.1 K.

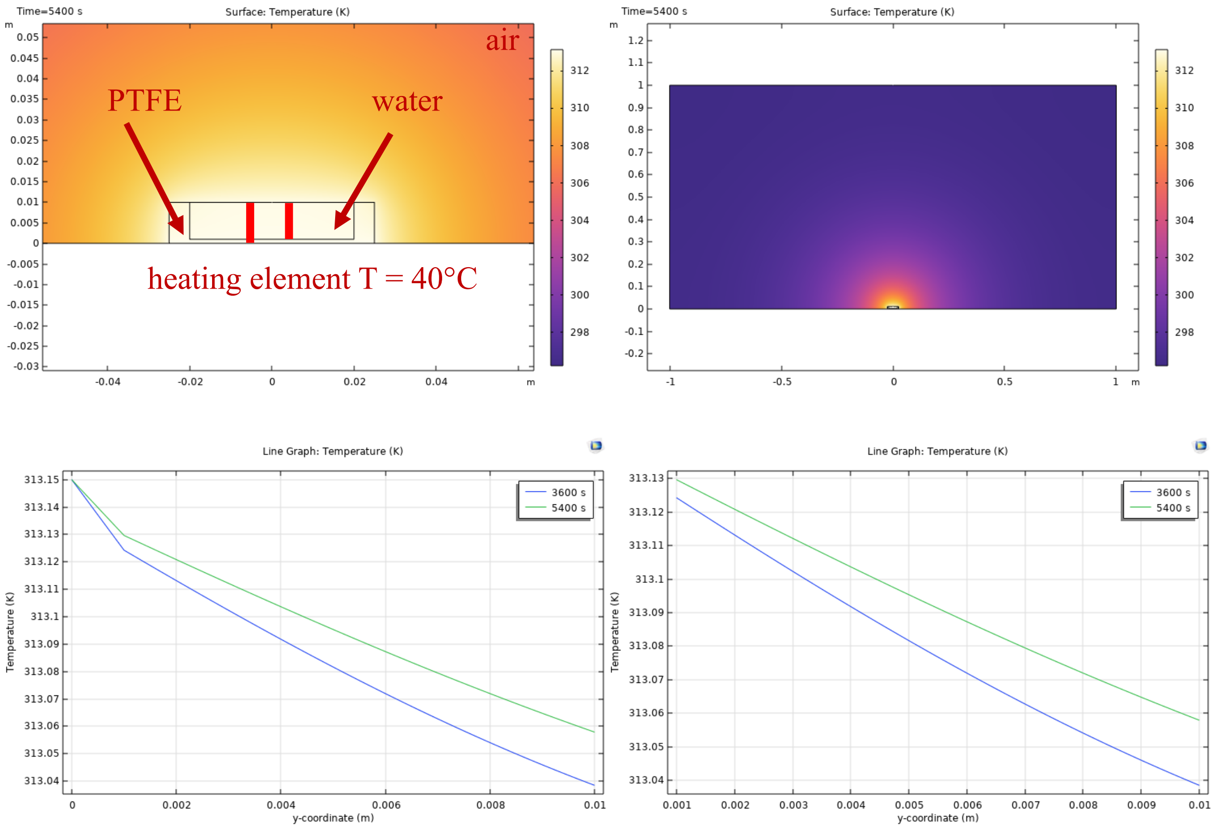

Figure S16. Calculations of the temperature gradient across the water subphase upon heating to 40 °C assuming the ambient temperature of 23 °C and Langmuir trough fully filled with water.

The differences in water temperature between the bottom and top of the trough reached only around 0.1 K.

Next, we calculated Rayleigh numbers for the system to establish if Rayleigh-Benard cells could form. The highest estimated number was around 1343 (for the situation depicted in **Figure S16**). This result corresponded to 60 min of equilibration. Upon additional 30 minutes of equilibration, the Rayleigh number decreases to around 309. The obtained Rayleigh values were below the critical value, *i.e.*, around 1700, above which convection cells appear[13]. Therefore, we neglected the small temperature gradient, as is generally accepted by Langmuir trough users.

The values used for calculating the Rayleigh numbers were: width of the trough 50 mm, depth of the water subphase 9 mm, and thickness of the PTFE layer between the heating element and water subphase 1 mm. Temperature-specific parameters were as follows:

- 40 °C: dynamic viscosity (μ) 0.00065 [Pa·s], density 992 [$kg \cdot m^{-3}$], volumetric expansion (β) 0.00021 [$°C^{-1}$], fluid thermal conductivity (λ) 0.635 [$W \cdot m^{-1} \cdot °C^{-1}$], fluid specific heat (Cp) 4170 [$J \cdot kg^{-1} \cdot °C^{-1}$],
- 20 °C: dynamic viscosity (μ) 0.001 [Pa·s], density 998 [$kg \cdot m^{-3}$], volumetric expansion (β) 0.00021 [$°C^{-1}$], fluid thermal conductivity (λ) 0.599 [$W \cdot m^{-1} \cdot °C^{-1}$)], fluid specific heat (Cp) 4182 [$J \cdot kg^{-1} \cdot °C^{-1}$].

References

(1) Hilles, H.; Monroy, F.; Bonales, L. J.; Ortega, F.; Rubio, R. G. Fourier-Transform Rheology

of Polymer Langmuir Monolayers: Analysis of the Non-Linear and Plastic Behaviors. *Adv. Colloid Interface Sci.* **2006**, *122* (1–3), 67–77. https://doi.org/10.1016/j.cis.2006.06.013.

(2) Morioka, T.; Kawaguchi, M. Surface Dilational Moduli of Polymer and Blended Polymer Monolayers Spread at Air-Water Interfaces. *Adv. Colloid Interface Sci.* **2014**, *214*, 1–16. https://doi.org/10.1016/j.cis.2014.10.003.

(3) Guzmán, E.; Liggieri, L.; Santini, E.; Ferrari, M.; Ravera, F. DPPC-DOPC Langmuir Monolayers Modified by Hydrophilic Silica Nanoparticles: Phase Behaviour, Structure and Rheology. *Colloids Surfaces A Physicochem. Eng. Asp.* **2012**, *413*, 174–183. https://doi.org/10.1016/j.colsurfa.2011.12.059.

(4) Guzmán, E.; Liggieri, L.; Santini, E.; Ferrari, M.; Ravera, F. Effect of Hydrophilic and Hydrophobic Nanoparticles on the Surface Pressure Response of DPPC Monolayers. *J. Phys. Chem. C* **2011**, *115* (44), 21715–21722. https://doi.org/10.1021/jp207713x.

(5) Guzmán, E.; Martínez-Pedrero, F.; Calero, C.; Maestro, A.; Ortega, F.; Rubio, R. G. A Broad Perspective to Particle-Laden Fluid Interfaces Systems: From Chemically Homogeneous Particles to Active Colloids. *Adv. Colloid Interface Sci.* **2022**, *302*. https://doi.org/10.1016/j.cis.2022.102620.

(6) Shi, S.; Russell, T. P. Nanoparticle Assembly at Liquid–Liquid Interfaces: From the Nanoscale to Mesoscale. *Adv. Mater.* **2018**, *30* (44), 1–22. https://doi.org/10.1002/adma.201800714.

(7) Aveyard, R.; Clint, J. H. Liquid Droplets and Solid Particles at Surfactant Solution Interfaces. *J. Chem. Soc. Faraday Trans.* **1995**, *91* (17), 2681–2697.

https://doi.org/10.1039/FT9959102681.

(8) Vargaftik, N. B.; Volkov, B. N.; Voljak, L. D. International Tables of the Surface Tension of Water. *Journal of Physical and Chemical Reference Data*. 1983, pp 817–820. https://doi.org/10.1063/1.555688.

(9) Zhang, J.; Pelton, R.; Deng, Y. Temperature-Dependent Contact Angles of Water on Poly(N-Isopropylacrylamide) Gels. *Langmuir* **1995**, *11* (6), 2301–2302. https://doi.org/10.1021/la00006a071.

(10) Zang, D. Y.; Rio, E.; Delon, G.; Langevin, D.; Wei, B.; Binks, B. P. Influence of the Contact Angle of Silica Nanoparticles at the Air-Water Interface on the Mechanical Properties of the Layers Composed of These Particles. *Mol. Phys.* **2011**, *109* (7–10), 1057–1066. https://doi.org/10.1080/00268976.2010.542778.

(11) Kralchevsky, P. A.; Nagayama, K. Capillary Interactions between Particles Bound to Interfaces, Liquid Films and Biomembranes. *Adv. Colloid Interface Sci.* **2000**, *85* (2), 145–192. https://doi.org/10.1016/S0001-8686(99)00016-0.

(12) Gaboriaud, F.; Volinsky, R.; Berman, A.; Jelinek, R. Temperature Dependence of the Organization and Molecular Interactions within Phospholipid/Diacetylene Langmuir Films. *J. Colloid Interface Sci.* **2005**, *287* (1), 191–197. https://doi.org/10.1016/j.jcis.2005.01.110.

(13) Koschmieder, E. L. *Bénard Cells and Taylor Vortices (Cambridge Monographs on Mechanics)*; Cambridge University Press, 1993.
